# Developmental transition of visual and nonvisual photoreception and circadian clock during smoltification in the eye and brain of Atlantic salmon

**DOI:** 10.1371/journal.pone.0349748

**Published:** 2026-05-21

**Authors:** Mariann Eilertsen, David W. P. Dolan, Rita Karlsen, Tom Ole Nilsen, Wayne I. L. Davies, Jon Vidar Helvik

**Affiliations:** 1 Department of Biological Sciences, University of Bergen, Bergen, Norway; 2 Department of Informatics, University of Bergen, Bergen, Norway; 3 Department of Molecular Biology, Umeå University, Umeå, Sweden; 4 School of Agriculture, Biomedicine and Environment, La Trobe University, Victoria, Australia; Karlsruhe Institute of Technology: Karlsruher Institut fur Technologie, GERMANY

## Abstract

Seasonal variation in photoperiod is an important cue that regulates changes in physiology and behavior during the anadromous lifestyle of Atlantic salmon. The parr-smolt transformation or smoltification, where fish migrate from rivers to the ocean, is promoted by an increased photoperiod in the spring. Photoperiodic information is transferred through the light-brain-pituitary axis, resulting in pituitary hormones stimulating changes related to this transition. The light environment is perceived by ocular photopigments in the rods and cones that convey image formation and via nonvisual photoreceptors entraining biological processes that synchronize with circadian and circannual light rhythms through the molecular clock mechanism. In this study, the dynamic expression of visual and nonvisual opsin genes and clock genes through smoltification were revealed by RNA sequencing. The results showed a dramatic transition of the teleost visual system by changes in expression of the tandem duplicated medium-wavelength-sensitive (*mws* or “green”) and long-wavelength-sensitive (*lws* or “red”) opsin genes during seawater migration, shifting the spectral sensitivity of the green opsins by up to 40 nm towards shorter wavelengths. Concomitantly, the expression of the *lws* opsin gene that forms a photopigment with the most extreme absorbance maximum was upregulated. Among the nonvisual opsins, the pineal-specific exorhodopsin was greatly upregulated in seawater, coinciding with an increased expression of important enzymes that dictate melatonin synthesis. Analyzing the components of the salmonid molecular clock expressed during smoltification showed that clock genes were dynamically expressed with changes in expression both related to changes in the photoperiod and the developmental transition from freshwater to seawater. The transcriptomic profile of the teleost brain through smoltification was shown to coincide with important genes that underpin the mammalian model of photoperiodism that drive summer and winter physiology, supporting common photoperiodic pathways that regulate seasonality in vertebrates.

## Introduction

The Atlantic salmon (*Salmo salar*) belongs to one of several salmonid classes, a collective group of teleost species with an anadromous lifestyle, with habitats that vary from freshwater rivers to the open sea, each exhibiting different photic environments. Specifically, the light environment quickly changes with depth as photons are absorbed and scattered by water molecules and components such as dissolved organic matter, chlorophyll in phytoplankton and suspended soil [1,2]. Importantly, shorter wavelengths in the light spectrum centered around 480 nm (perceived as “blue”) penetrate deepest in the ocean, while even shorter wavelengths (i.e., ultraviolet light and violet light to a lesser degree) and longer wavelengths (perceived as “red”) are limited to the upper water column [3]. As most rivers and estuaries are not as deep as the open ocean, the extremes of the light spectrum are far less devoid of shorter and longer wavelengths in comparison, although some light filtering is observed. Thus, the changing light conditions in the aquatic environment sets critical visual constraints and teleost fish are required to change their visual capacity in response to these marked shifts in spectral composition, especially during key developmental transitions [4,5].

The detection of light (i.e., photoreception) is primarily mediated by photoreceptors that consist of an opsin protein bound to a light-sensitive chromophore which together are located in the outer membrane of photoreceptor cells [6]. In essence, opsins may be broadly divided into different classes of visual opsins that are located in the rods and cones of the retina and nonvisual opsins that are more widely expressed, e.g., different retinal layers, the pineal organ, the deep brain and most teleost tissues [7–9]. For visual photoreception, low light intensities are detected by rods that express rod opsin (RH1, with a spectral peak of absorbance (λ_max_) value ranging from 460 to 530 nm), while cone opsin-based photopigments mediate bright light and color vision via one of four classes based on the peak spectral sensitivities. Specifically, their peak absorbances discretely lie within the ultraviolet (UV) to violet (SWS1, λ_max_ 350–440 nm), blue (SWS2, λ_max_ 430–470 nm), green (RH2, λ_max_ 460–530 nm) and red (LWS, λ_max_ 520–575 nm) regions of the light spectrum [10]. Recently, the complexity and spectral sensitivity of the visual opsins in Atlantic salmon were characterized, identifying several duplicated orthologues for all five visual opsin classes, except for presence of a single *sws2* gene [11]. However, based on sequence analysis, only 11 of the visual opsins appeared functional, with the tandem duplicated *rh2* class predicted to have the largest range in peak absorbance that differed almost 40 nm [11].

Over the past decade, a greater complexity in the discovery and biological relevance of nonvisual opsins has been observed, with many nonvisual photopigments (e.g., OPN3, OPN4 and OPN5 especially) being identified as key players in the synchronization of circadian and circannual rhythms, and multiple behavioral and physiological responses [8,12]. Phylogenetically, nonvisual opsins in vertebrates may be divided into several families, including exorhodopsin (EX-RHO), vertebrate ancient (VA) opsin, OPN3 (opsin 3, also called panopsin/encephalopsin), OPN4 (opsin 4, also known as melanopsin), OPN5 (opsin 5, also known as neuropsin), peropsin (RRH), retinal G protein-coupled receptor (RGR) opsin, teleost multiple tissue (TMT) opsin, parietopsin and parapinopsin [13] with the addition of more recently discovered OPN6, OPN7, OPN8 and OPN9, some of which appear to be teleost-specific and exhibit differing spectral biochemical properties [8]. Like visual photopigments, genetic variation in nonvisual opsin sequences, especially those in fish, has resulted in spectral absorbance profiles that range from approximately 350 nm to almost 550 nm, with many nonvisual photopigments peaking around 450–500 nm [9]. Of particular interest is the extra whole genome duplication in salmonids [14,15], which provides an even greater level of complexity in the nonvisual opsin repertoire in Atlantic salmon, with an array of at least 42 opsin genes [16,17].

The phenotypic plasticity of photoreception, especially in fish species, resides within the complexity of functional opsins present in the genome, their ontogenetic expression patterns and their diverse spectral properties [4]. Ontogenetic changes in opsin expression have been shown in many teleosts [18–21], including Atlantic cod (*Gadus morhua*), which has a long larval period accompanied by the presence of a pure cone retina and the development of rods in the transition to the juvenile stage [22,23]. Interestingly, the expression profile of *rh2* cone opsin genes in cod larvae decreased dramatically as rod opsin expression appeared [23]. However, exposure to diverse spectrally different light environments did not change cone opsin expression in cod larvae, which thereby indicates that larval plasticity is controlled by a developmental genetic program rather than the light environment, at least in this particular species [24]. In salmonids, the loss of UV-sensitive cones that express *sws1* is related to a migration from shallow freshwater rivers (with abundant UV light) to deeper and darker oceanic waters, thus representing a shift in opsin expression that directly correspond to a developmental transition that is accompanied by changes in the photic environment [25,26]. Despite ontogenetic changes in both *rh2* and *lws* opsin classes of Atlantic salmon not yet being described, studies in other teleosts have shown that green and red opsins are differentially expressed both temporally and spatially [20,27]. Further, reports demonstrate that thyroid hormone (TH) and retinoic acid (RA) are critical for the differential expression of these tandem duplicated opsins when determining the dorsal-ventral axis in the retina [28]. Ontogenetic changes in nonvisual opsin expression are less explored, but a study on hatching mechanism in Atlantic halibut (*Hippoglossus hippoglossus*) has suggested that hatching is under the direct control of transient deep brain photoreceptors [29]. Only a few studies have investigated molecular shifts in nonvisual opsin expression that correspond to changes in the photic environment. For example, in the viviparous four-eyed fish (*Anableps anableps*) the regionalized expression of ocular nonvisual opsins altered when juvenile fish were raised in clear water instead of natural muddy water, thus indicating that different light conditions are able to modulate nonvisual opsin expression [30]. In Atlantic salmon, the expression of nonvisual opsins is found in different retinal layers and in the brain [31,32], with RNA sequencing approaches showing that nonvisual opsins are present in early development (e.g., before hatching) with an increase in expression towards first feeding [17]. However, differing opsin expression profiles that reflect changes in either the photic environment or a particular developmental transition have not been described.

Atlantic salmon has a seasonally driven anadromous lifestyle, where migration from freshwater rivers into the ocean is timed to coincide with the longer photoperiod experienced during the spring. This seasonal information is mediated through the light-brain-pituitary axis, which results in the release of pituitary hormones that stimulate physiological changes related to the transition from freshwater to saltwater, termed the parr-smolt transformation or smoltification [33,34]. Importantly, photoperiodic shifts such as changing from short to long days, lead to increased levels of thyroid-stimulating hormone (*tsh*β*b*) in the pituitary and subsequent upregulation of deiodinase (*dio2b*) in the brain together with increased plasma growth hormone (*gh*) and other well-established indicators of smolt development, thereby supporting a common photoperiodic regulation of seasonality in vertebrates [35]. In the mammalian model of photoperiod-driven seasonal responses (termed photoperiodism) a photoperiodic switch between genes involved in the circadian clock mechanism is suggested to drive winter and summer physiology [36], but information about the mechanism in teleosts is greatly limited. Further, information on how photoperiodic cues are perceived and transmitted during parr-smolt transformation is scarce. Hence, the study aims to address these unanswered questions by applying transcriptomic approaches to analyze the gene expression in the eye and brain during smoltification. It has been shown that components of the molecular clock have different expression patterns in the parr brain when developing fish are exposed to either long or short photoperiods, thereby indicating that the clock mechanism is day-length dependent [37]. Further, RNA sequencing of gills through smoltification has shown that 30 clock genes are differentially expressed during this transformation [38]. Thus, this current study will also analyze several key clock genes to reveal any modulation in their expression profiles through photoperiodic shifts that relate to migration from freshwater to seawater. The results showed comprehensive expression profiles of opsin genes and clock genes during the parr-smolt transformation, revealing that visual opsin genes in the eye are far more dynamic than earlier reported for salmonids. Further, analysis of elements of the mammalian model of photoperiodism revealed an expression profile of genes in accordance with the photoperiodic switch that drives the winter and summer physiology.

## Materials and methods

### Ethical statement

The study was performed on a sibling group of Atlantic salmon (*Salmo salar*) from Mowi, Tveitevågen, Norway. Euthanasia and subsequent sacrifice of fish and the provision of eggs and sperm were conducted by an on-site aquaculture farmer according to the Regulations on Slaughterhouses and Production Facilities for Aquaculture Animals (FOR-2014-12-15-1831). Unfertilized eggs and sperm were brought to the High Technology Center, Bergen, Norway, where both fertilization and fish rearing occurred in a facility (University of Bergen) approved by the Norwegian Food Safety Authority (VSID2135). Fish did not undergo handling except for euthanasia and, therefore, special approvals were not required according to Norwegian national legislation as stated in the Norwegian Animal Welfare Act (LOV-2015-06-09-16-65) and Regulations on the Use of Animals in Experiments (FOR-2017-04-05- 451), given by the EU for animal experiments (Directive 2010/63/EU). All fish were euthanized on-site with an overdose of metacaine (MS-222, MSD Animal Health, Netherlands) before further handling.

### Experimental design and sampling

Details of the 557 days experiment and sampling points are given in S1 Fig. The fertilized eggs were reared in egg incubators with a light:dark (LD) cycle of 14:10 (light switched off and on at 22:00 and 08:00, respectively) from fertilization to first feeding with white LED as described in [17]. Three months before sampling of the parr stage, the lights were changed to two ceiling lamps containing four fluorescent tube lights (GE Starcoat T5 Long Last 1449 mm Fluorescent Tube 49W, 3000K Warm white (General Electric, MA, USA)) for the rest of the experiment. The feeding fry to parr stage were kept in a LD cycle of 20:4 until they reached a size of approximately 11 cm. Photoperiodic induction of the parr-smolt transformation was conducted by exposing the parr to a LD 12:12 photoperiod (light switched off and on at 20:00 and 08:00, respectively), which mimics a winter light signal for 6 weeks, followed by a full 24 h daylength (LD 24:0) to resemble the summer light environment. This latter light period of LD 24:0 was subsequently maintained until the end of the experiment. During the experiment the fish were kept in black tanks (60 cm in diameter x 70 cm high). The fish were feed with Ewos (Norway) freshwater diet with the appropriate feed according to the size of the fish, by automatic feeders following the light period. The temperature from fertilization to first feeding was 6.0 ± 0.5°C which was gradually increased after first feeding to 11.5°C. The temperature was held constant for the rest of the experiment, as the temperature for avoiding the temperature as a zeitgeber, and it was determined to be 11.5 ± 0.3°C prior to seawater transfer, 11.1 ± 0.13°C after this transition. The transition from fresh to sea water was done gradually by a change of the inlet water from fresh to seawater, generating full seawater within a few hours. Five samplings were conducted through the parr-smolt transformation with the four first stages in freshwater and the fifth stage in seawater. At each stage, four fish were euthanized, weighed and the fork length measured, further, brains and left eyes were carefully dissected and individually snap frozen in liquid nitrogen, before storage at – 80°C. The following stages were sampled starting at 12:00: i) parr before the application of a winter signal (Parr, zeitgeber time (ZT) ZT8); (ii) parr reared and exposed to a winter signal for 6 weeks (ParrWS, ZT4); (iii) mid-smolt 22 days after transition to the summer signal (MidSmolt, no ZT); (iv) peak-smolt 51 days after applying the summer signal (PeakSmolt, no ZT); and (v) post-smolt approximately one month in seawater (PostSmolt, no ZT) (see S1 Fig). Note that eyes were unfortunately not sampled at i) parr before the application of a winter signal (Parr), only brains. Weights (g), fork lengths (cm) and condition factors (where K-factor = (100*weight)/(length^3)) were plotted with standard deviation (S2 Fig) to show that the K-factor values, a well-established indicator of smolt development, were consistent with the literature and decreased during smoltification [39,40].

### RNA extraction

Four brains and eyes per stage were immersed in prechilled (−80°C) RNA later ICE (Invitrogen, Carlsbad, CA, USA) for at least 48 h at −20°C. After treatment, brains were individually inspected to confirm that the pineal and pituitary glands were intact, and the lenses of the eyes were removed. Total RNA was isolated from individual whole brains and eyes using TRI-reagent (Sigma, St. Louis, MO, USA) and the brains and eyes were divided into pieces less than 100 mg before total RNA isolation, following manufacturer’s instructions. The isolation was performed separately on each piece and prior to the precipitation step, lysates originating from the same brain or eye were pooled to ensure that the final RNA sample represented the entire individual brain or eye. After purification, a nanodrop ND-1000 spectrophotometer (Thermo Fisher Scientific, Waltham, MA, USA) was used to measure the RNA quantity and quality before and after DNase I treatment (TURBO DNA-free Kit, ThermoScientific, Waltham, MA, USA). Prior to RNA sequencing, RNA integrity was checked using an Agilent2100 Bioanalyzer with RNA 6000 Nano Kit (Agilent Technologies, CA, USA). All RNA integrity numbers (RIN) were determined to be between 8.4-10 and deemed valid for further RNA processing.

### RNA sequencing

For RNA sequencing total RNA samples were submitted to the Genomics Core Facility at the University of Bergen (Bergen, Norway). Samples were processed using Illumina TrueSeq Stranded mRNA Sample Preparation Kits according to the manufacturers protocol and sequenced on the Illumina HiSeq 4000 platform (Illumina, San Diego, CA, USA. Sequencing generated a mean of 57 million 75 bp paired end reads per sample, which is within the recommendations from Illumina (https://knowledge.illumina.com/library-preparation/rna-library-prep/library-preparation-rna-library-prep-reference_material-list/000001243). All sequencing data were deposited to the European Nucleotide Archive (Accession number PRJEB103069). In addition, previously generated RNA sequencing data [17] were included and analyzed, to expand the timeline and give an initial developmental profile of Atlantic salmon. Specifically derived from this developmental series of whole Atlantic salmon individual samples were obtained at 255 dd (around the eye pigmentation stage (n = 6)), 379 dd (before hatching (n = 6)), 555 dd (after hatching (n = 6)) and 690 dd (before first feeding (n = 8)). The developmental series of larvae and alevins was sampled (ZT2) and snap frozen in liquid nitrogen and total RNA was extracted using the same protocols as described for brains and eyes. RNA sequencing of the developmental series was conducted at Genomics Core Facility at the University of Bergen (Bergen, Norway) as described above, see [17] for details.

### RNA sequencing analysis

After RNA sequencing, all results were trimmed by applying Trimmomatic software version 0.38 [41] and aligned to the published Atlantic salmon reference genome (http://ftp.ensembl.org/pub/release-106/gtf/salmo_salar/) by using STAR version 2.7.0 [42]. Output files were processed by Samtools version 1.6 [43] and counts were generated using HTSeq version 0.11.2 [44]. Two arrays of normalized count files were generated by DESeq2 version 1.26.0 [45] namely (i) normalized counts of the eyes through the parr-smolt transformation (S1 Table) and (ii) normalized counts of the brains through the parr-smolt transformation (S2 Table). The variability in the data was visualized through two Principal Component Analysis (PCA) plots (S3 Fig) generated by DESeq2, using the default ntop = 500 top features by variance, showing eye and brain samples, respectively. DESeq2 was also used to perform differential gene expression analyzes applying the Wald statistical model with an adjusted p-value was set to < 0.05. In the analyzes genes with counts less than 10 per comparison were not included. The differential expression analysis compared all sampling stages against each other. For the eye samples six comparison were done (ParrWS vs MidSmolt, ParrWS vs PeakSmolt, ParrWS vs PostSmolt, MidSmolt vs PeakSmolt, MidSmolt vs PostSmolt, PeakSmolt vs PostSmolt) and for the brain samples ten comparisons were done (Parr vs ParrWS, Parr vs MidSmolt, Parr vs PeakSmolt, Parr vs PostSmolt, ParrWS vs MidSmolt, ParrWS vs PeakSmolt, ParrWS vs PostSmolt, MidSmolt vs PeakSmolt, MidSmolt vs PostSmolt, PeakSmolt vs PostSmolt). Annotated Ensembl GeneIDs of Atlantic salmon Ssal_v3.1 were used in the analyzes, see annotations in [11] for the visual opsin. Where possible, opsin gene names and their spectral sensitivities will be referred to by the use of standard photopigment nomenclature conventions and their specific wavelengths. In some cases to improve understanding, however, some opsins may be stated in terms of color when referring to the region of the light spectrum that their resultant photopigments are sensitive to, although it is acknowledged that color is perceived and is a concept that is strongly based on human psychophysical principles (e.g., blue, green and red opsins refer to photopigments that are short-, medium- and long-wavelength-sensitive). Further, see [46] for annotations of clock genes, circadian rhythm genes and enzymes of the melatonin biosynthesis pathway. Updated annotations of nonvisual opsins genes in Atlantic salmon, genes involved in the metabolism of TH and RA, and genes derived from the mammalian model of photoperiodism are listed in S3 Table. Graphs visualizing the normalized counts and PCA plots were generated using the ggplot2 v3.3.6 package [47] in Rstudio [48] and fold-changes were calculated by comparing logfold2 change between an average normalized count for one group against another.

## Results

### Dynamics of visual opsins gene expression in early development

**Fig 1:**
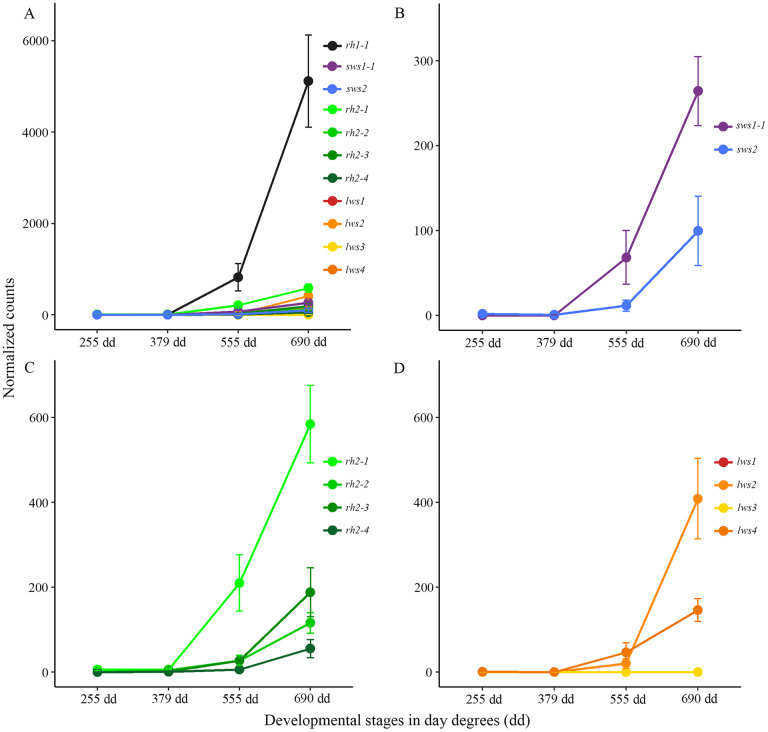
Visual opsin gene expressions at early developmental stages before first feeding. The graphs show normalized counts in the whole alevin with standard deviation at four developmental stages before first feeding. **A)** All 11 functional rod (black line) and cone (colored lines) opsins plotted together, showing that rod opsin (*rh1−1*) was expressed at the highest level compared to all cone opsin subclasses. **B)** Plotting of the short-wavelength-sensitive opsins shows that the expression *of sws1−1* increased during hatching, with *sws2* being substantially expressed only before first feeding at 690 dd. **C)** Medium-wavelength-sensitive opsins are all expressed before first feeding, with *rh2−1* expression increasing during hatching, while the other *rh2* genes showed substantial expression levels around 690 dd. **D)** Among the long-wavelength-sensitive opsins, both *lws2* and *lws4* were expressed before first feeding, with *lws2* being the most prominent. Both *lws1* and *lws3* exhibited no expression during early development.

RNA sequencing was used to study visual opsin expression in whole Atlantic salmon larvae and alevins, ranging from the eye pigmentation stage to before first feeding. The results showed little or no expression of visual opsins genes before hatching, but the expression levels of all visual opsin classes started to increase during the transition of hatching (370 dd and 555 dd), with substantial expression towards first feeding (690 dd) (Fig 1). The expression of rod opsin (*rh1–1*) was most prominent (around 73% at 690 dd) (Fig 1A), followed by the subsequent primary cone classes: *rh2–1* (a green or medium-wavelength-sensitive opsin) (Fig 1C), *lws2* (red or long-wavelength-sensitive) (Fig 1D), and *sws1–1* (UV-sensitive opsin) (Fig 1B). A single blue opsin class (*sws2*) (Fig 1B), three out of four green opsins (r*h2-2*, *rh2–3* and *rh2–4*) (Fig 1C), and one out of four red opsins (*lws4*) (Fig 1D) were also expressed at a lower level, while two other red opsins (*lws1* and *lws3*) (Fig 1D) were not expressed before first feeding.

### Dynamics of visual opsins expressed in the eyes during the parr-smolt transformation

Differential gene expression analyzes showed that many genes were differentially expressed in the eye through smoltification, with an adjusted p-value set to < 0.05. Table 1 shows the total number of differentially expressed genes (DEGs) across the various stages of smoltification, with visual opsin genes being indicated. Also, see S4 Table for details, listing DEGs (between all stages) among the visual and nonvisual opsin genes and genes important in the metabolism of TH and RA, the latter being two molecular components that are involved in regulating the differential expression of tandem duplicated opsins, such as *rh2* and *lws* subclasses [28].

**Table 1:** The number of differentially expressed genes (DEGs) (adjusted p-value < 0.05) in the eye of Atlantic salmon.

	ParrWS	MidSmolt	PeakSmolt	PostSmolt
ParrWS	–	611 (2)	1733 (6)	5711 (6)
MidSmolt	–	–	560 (6)	813 (4)
PeakSmolt	–	–	–	5665 (4)
PostSmolt	–	–	–	–

The number of visual opsins identified among the total DEGs are indicated in parentheses.

**Fig 2:**
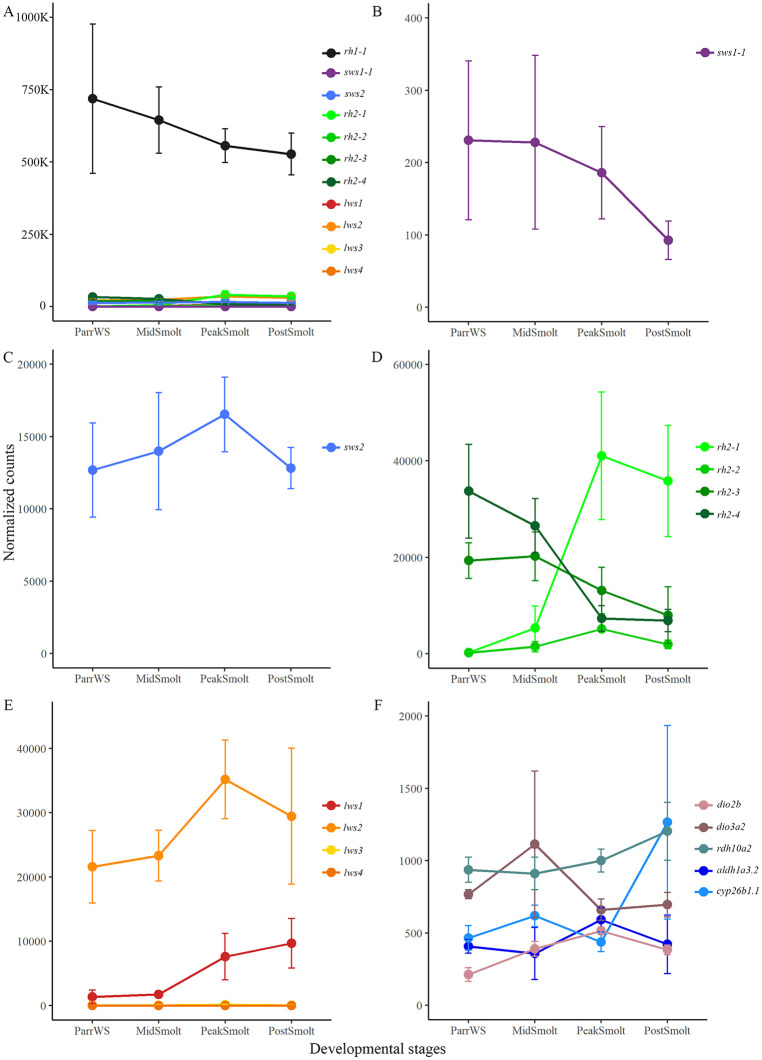
Visual opsin expression in the eye during the parr-smolt transformation. The graphs show normalized counts with standard deviation at four sampling points through smoltification. **A)** All rod (black line) and cone (colored lines) visual opsins are plotted together showing that the expression of *rh1-1* was dominant in the eye through smoltification. **B)** Short-wavelength-sensitive-1 opsin (*sws1-1*) significantly decreased in expression from ParrWS to PostSmolt. **C)** Expression of *sws2* was relatively stable through smoltification. **D)** Medium- wavelength-sensitive opsins demonstrated dynamic expression patterns through smoltification, with *rh2-1* being the gene with prominent expression in the seawater phase at PostSmolt, with *rh2-2* being lowly expressed at all stages, with *rh2-3* and *rh2-4* being dominantly expressed at ParrWS. **E)** Among the long-wavelength-sensitive opsins, *lws1* increased in expression towards the seawater phase, *lws2* was highly expressed throughout smoltification with a peak at PeakSmolt, whereas *lws3* and *lws4* were both lowly expressed. **F)** Selected ventralizing factors involved in the metabolism of thyroid hormone (TH) (namely *dio2b* and *dio3a2*) and retinoic acid (RA) (specifically *rdh10a2*, *aldh1a3.2* and *cyp26b1.1*) were dynamically expressed with maximum expression levels towards the seawater phase.

All 11 functional visual opsins were expressed in the eye during the parr-smolt transformation, with many exhibiting dynamic expression profiles through smoltification (Fig 2). In addition, these visual opsin smoltification expression patterns were compared to selected genes important in the metabolism of RA and TH to determine if these latter ventralizing factors coincided with the differential expression of tandem duplicated *rh2* and *lws* opsin gene classes. As expected, rod opsin continued to be the most prominently expressed opsin and held around 85% of the total normalized counts for the 11 visual opsin genes in the eye through smoltification (Fig 2A). However, *rh1−1* was not differentially expressed during smoltification (S4 Table) and had an average normalized count of over 600 000. By contrast, *sws1−1* exhibited an over 1-fold decrease in expression from ParrWS to PostSmolt and was differentially expressed between these two stages (Fig 2B and S4 Table). Blue (*sws2*) opsin showed a relatively constant expression through smoltification (with an average of approximately 14 000 normalized counts between the stages) and was only differentially expressed between PeakSmolt and PostSmolt with a small fold decrease in expression (Fig 2C and S4 Table). Among the green opsins (Fig 2D), the expression of *rh2−1* differentially increased greatly during smoltification and by 7-fold from ParrWS to PeakSmolt (S4 Table), with an average normalized count of over 40 000 at PeakSmolt. Compared to the other green opsins, *rh2−2* exhibited low expression levels with an average of around 2000 normalized counts for the four stages but was differentially expressed between several sampling conditions (Fig 2D and S4 Table). For example, *rh2−2* was lowly expressed at ParrWS, and had an almost 5-fold increase in expression between ParrWS and PeakSmolt. For both *rh2−3* and *rh2−4* the expression decreased though smoltification, with high expression levels in ParrWS (around an average of 20 000 and 30 000 normalized counts, respectively) and low in PostSmolt (around an average of 7000 and 8000 normalized counts, respectively), totaling over 1-fold (*rh2−3*) and 2-fold (*rh2−4*) decrease between these two outer stages (Fig 2D), however the *rh2−3* was not significantly differentially expressed at any stage (S4 Table). Regarding red opsin expression, *lws2* showed the highest level of expression, which was also observed at early developmental stages, with the greatest level of *lws2* observed at PeakSmolt (approximately 35 000 normalized counts), upregulated approximately 1-fold from ParrWS to PeakSmolt (Fig 2E). Although not expressed before first feeding, the expression of *lws1* increased differentially through smoltification and had almost a 3-fold increase from ParrWS to PostSmolt (from around an average of 1300–9700 normalized counts), yielding a 2-fold increase between MidSmolt and PeakSmolt (Fig 2E and S4 Table). In contrast to *lws1* and *lws2*, the expression levels of *lws3* and *lws4* were very low throughout smoltification. Among the genes that function as ventralizing factors (see Fig 2E for selected genes based on expression levels and S4 Fig for all members), the deiodinase members, which are involved in the metabolism of TH, peaked at MidSmolt (i.e., *dio3a2*) and PeakSmolt (specifically *dio2b* were differentially expressed with an over 1-fold change from ParrWS to PeakSmolt) stages. Whereas genes that are involved in the metabolism of RA showed an increase during smoltification, for example, retinaldehyde dehydrogenase (*aldh1a3.2*) had highest expression at PeakSmolt with a significant increased from ParrWS to PeakSmolt, and a member of the cryptochrome P450 class, *cyp26b1.1* was significantly peaking at PostSmolt with a 1,5- fold change from PeakSmolt to PostSmolt (Fig 2E and S4 Table). Interestingly, peaks of *dio2b* and *aldh1a3.2* at PeakSmolt coincided with peaks in *rh2−1* and *lws1* (Figs 2D-2F).

### The dynamics of visual opsin expression reflect significant spectral shifts in ocular photopigments of Atlantic salmon

The predicted absorbance maxima (i.e., λ_max_ values) of visual photopigments found in Atlantic salmon were previously been determined using manual examination of known and well-understood tuning sites and molecular dynamics simulations-based modeling approaches [11].

**Fig 3:**
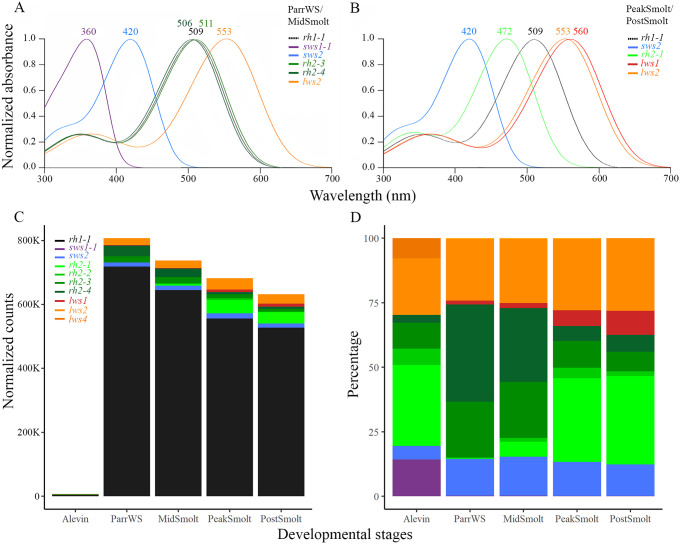
Spectral sensitivity dynamics of visual photopigments. The spectral profiles of visual photopigments are based on previously determined absorbance maxima (i.e., λ_max_ values) based on inferred predictions [11]. Note that these λ_max_ values applied a standard A_1_-based rhodopsin template in the evaluation as salmonid ocular photoreceptors (and the photopigments expressed therein) predominantly utilize a retinal chromophore derived from vitamin A_1_ [26]. **A)** Both ParrWS and MidSmolt had tetrachromatic vision with expression of *sws1−1* (λ_max_ of 360 nm), *sws2* (λ_max_ of 420 nm), *rh2−3* (λ_max_ of 511 nm) and *rh2−4* (λ_max_ of 506 nm) and *lws2* (λ_max_ of 553 nm). **B)** In PeakSmolt and PostSmolt the vision became trichromatic as *sws1−1* expression diminished. Great shifts in expression levels among the green photopigments were observed, expression levels of *rh2−3* (λ_max_ of 511 nm) and *rh2−4* (λ_max_ of 506 nm) were downregulated while *rh2−1* (λ_max_ of 472 nm) was greatly upregulated, accounting for peak spectral sensitivity change of up to almost 40 nm. In addition, the red photopigment *lws1* (λ_max_ of 560 nm) had an increase in expression level shifting the spectral sensitivity 7 nm towards the longer wavelengths. **C)** The total number of normalized counts of visual opsins in whole alevins (data including whole body) and the eyes through smoltification visualized as bars, illustrating high expression levels of rod opsin, accounting for over 80% of the total counts for visual opsin genes during smoltification, with the highest level in ParrWS (approximately 89%). **F)** The dynamics of cone opsin genes calculated for each stage, given by the percentage of the total normalized counts of visual opsins not including *rh1−1*.

As such, the spectra for visual photopigments expressed at different developmental as shown in Fig 3. In general, the total number of normalized counts for visual opsins in the eye during smoltification was highest in ParrWS exposed to a photoperiod of 12:12 (i.e., 12 h less of simulated daylight compared to the other stages), especially the *rh1−1* gene expressed in rods responsible for dim light vision, and the expression decreased as the photoperiod was changed to continuous light (LD 24:0) (Fig 3E). At the alevin stage just before first feeding, all visual photopigment genes from *sws1−1* (with a λ_max_ at 360 nm) to *lws2* (with a λ_max_ at 553 nm) were expressed, except for *lws1* (560 nm) and *lws3* (543 nm), however note the low number of normalized counts apparent in a whole alevin compared to eyes through smoltification (Fig 3C). Among the normalized counts for visual opsins rod opsin counted for around 73%, followed by *rh2−1*, *lws2* and *sws1−1*, visualized in Fig 3D by percentage of the total normalized counts of visual opsins not including *rh1−1*. Overall, both ParrWS and MidSmolt stages exhibited similar expression profiles, notable with expression of *sws1−1* (λ_max_ of 360 nm) and both *rh2−3* (λ_max_ of 511 nm) and *rh2−4* (λ_max_ of 506 nm) were highly expressed in ParrWS and MidSmolt, together with *lws2* (λ_max_ of 553 nm) (Fig 3A, 3F, 3E). Surprisingly, in addition to the declining *sws1−1* expression, at both PeakSmolt and PostSmolt stages, there were shifts in green photopigment gene expression, from *rh2−3* (λ_max_ of 511 nm) and *rh2−4* (λ_max_ of 506 nm) to *rh2−1* (λ_max_ of 472 nm), accounting for peak spectral sensitivity change predicted to be almost 40 nm towards the shorter wavelengths (Fig 3B, 3E, 3F). Despite the significant short-wavelength shift in *rh2*-based photopigments, Atlantic salmon exhibited a small long-wavelength shift of predicted 7 nm in the *lws* class of photopigments, a change derived from an increase in expression of *lws1* (λ_max_ of 560 nm) in addition to the relatively high and stable expression of *lws2* (λ_max_ of 553 nm) during smoltification (Fig 3D, 3E, 3F). Of note, blue opsin (*sws2*) had a relatively stable expression during smoltification, while *lws3* (λ_max_ of 546 nm) was not substantially expressed at any of the developmental stages examined.

### Transcriptomic changes of nonvisual opsins in the eye and brain during parr-smolt transformation

There were eleven nonvisual opsin genes among the DEGs in the eye during smoltification (S4 Table) and their dynamics are shown in S5 Fig. Notable, members of the retinal G protein-coupled receptor opsin (*rgr*) had high expression level compared to other nonvisual opsins in the eye during smoltification. The highest expression level was observed for *rgrb2* with an average of 10 000 normalized counts at MidSmolt and a downregulation in expression at PeakSmolt, with an over 1-fold decrease in expression between MidSmolt and PeakSmolt and an over 1-fold increase between PeakSmolt and PostSmolt (see S4 Table for details). Further, *opn6a1* had highest expression at ParrWS, before the expression level decreased towards the seawater phase by almost 1-fold change (S5 Fig). In addition, differential gene expression analyzes, with an adjusted p-value set to < 0.05, were done for the brain comparing all stages through smoltification and Table 2 reveals many DEGs in the brain, including the number of nonvisual opsins among the DEGs (see also details in S5 Table).

**Table 2:** The number of differentially expressed genes (DEGs) (adjusted p-value < 0.05) in the brain.

	Parr	ParrWS	MidSmolt	PeakSmolt	PostSmolt
Parr	–	17035 (7)	12968 (7)	4354 (3)	17895 (14)
ParrWS	–	–	88 (0)	6505 (3)	2116 (7)
MidSmolt	–	–	–	912 (0)	326 (1)
PeakSmolt	–	–	–	–	7238 (5)
PostSmolt	–	–	–	–	–

The number of nonvisual opsins identified among the total DEGs are indicated in parentheses.

**Fig 4:**
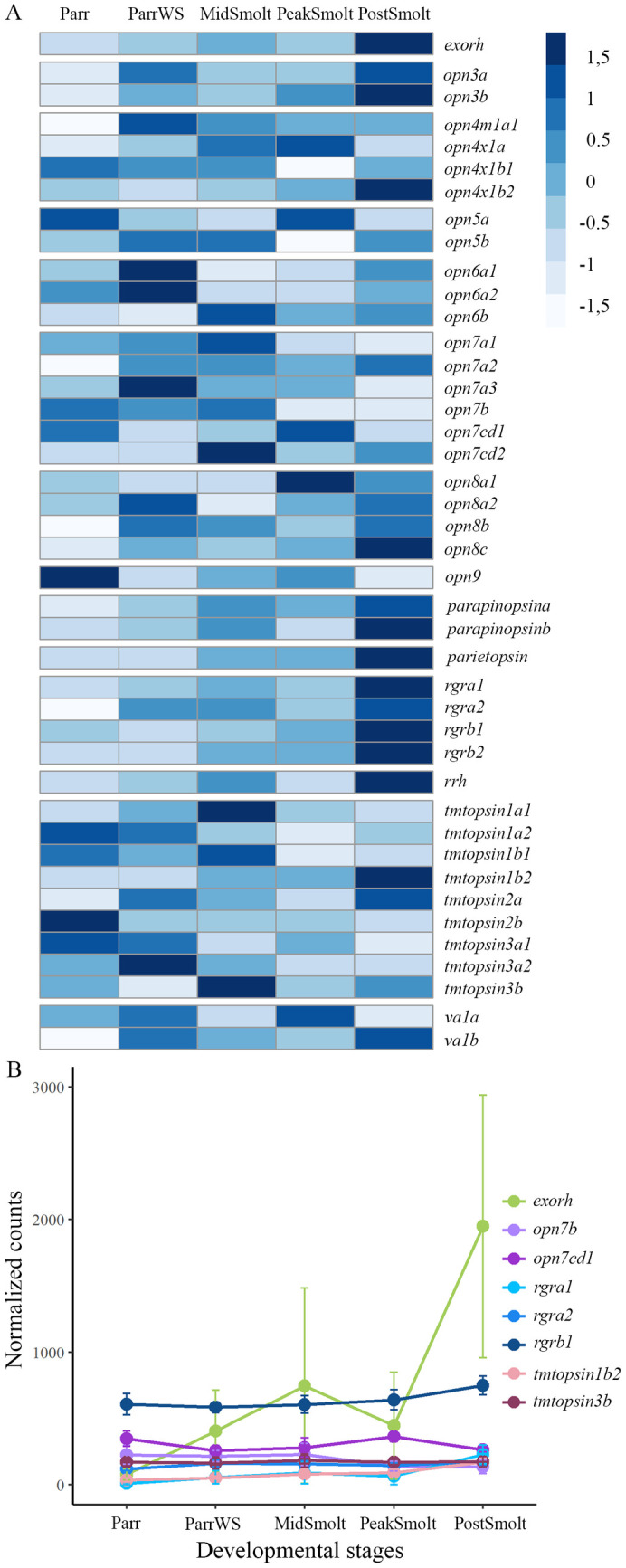
The dynamics of nonvisual opsins during smoltification. **A)** Heatmap of the normalized counts of all nonvisual opsins in the brain, scaled by row, demonstrates an increase in several of the opsins towards the seawater stage. **B)** The graphs show normalized counts with standard deviation at five sampling points in the brain through smoltification for the eight nonvisual opsins with normalized counts greater than 100. Exorhodopsin (*exorh*) had the highest expression among the opsins in the brain, followed by retinal G protein-coupled receptor opsin (*rgrb1*).

The expression profiles of 42 nonvisual opsins in the Atlantic salmon brain during smoltification showed that several of the opsins have increased expression towards the seawater phase (Fig 4A). However, only eight nonvisual opsins had a count over 100 and their expression dynamics were plotted in Fig 4B and among the plotted genes, *tmtopsin3b* was the only gene not significantly differentially expressed (S5 Table). Interestingly, the pineal-specific exorhodopsin had the most prominent expression level in the brain, followed by *rgrb1*, and had an almost 5-fold significant increase in expression level from Parr to PostSmolt (S5 Table).

**Fig 5:**
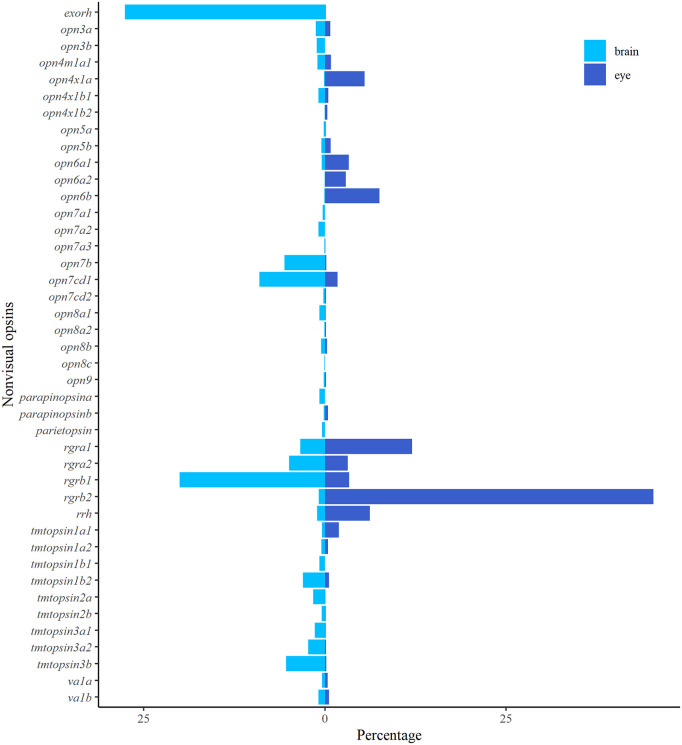
Differential expression profiles of nonvisual opsins in the eye and brain of Atlantic salmon. Bar chart of the nonvisual opsins plotted by percentage in the eye and brain. The total number of normalized counts at the four sampling points (ParrWS to PostSmolt) for the nonvisual opsins was set to 100 percent for each tissue and the distribution among the different opsins was plotted back-to-back. In the brain *exorh* was the most abundant opsins, followed by genes in the *rgr* class. In the eye, genes in the *rgr* class were highly expressed, with *rgrb2* counting for approximately 45%.

Further, the distribution of normalized counts for nonvisual opsins were visualized by percentage to reveal the differential expression profile between the eye and brain over the course of smoltification as a total (Fig 5). The results showed that in the brain *exorh* and *rgrb1* (with approximately 28% and 20% of the total nonvisual opsin counts, respectively) were the most abundant genes, followed by genes in the *opn7* class (e.g., *opn7b* and *opn7 cd1* at approximately 6% and 9%, respectively) and *tmtopsin* class (e.g., *tmtopsin3b* at approximately 5%). In the eye, genes in the *rgr* class held 65% of the normalized counts, with a high expression level of *rgrb2* (around 45% of the counts). In addition, genes in the *opn6* class held in total approximately 14% of the normalized counts, *rrh* approximately 6% and *opn4x1a* above 5%. Interestingly, *rgrb2* and not *rgrb1* was highly expressed in the eye, indicating a sub-functionalization of the two genes.

### Dynamics of clock genes and outputs of the circadian clock mechanism during smoltification

**Fig 6:**
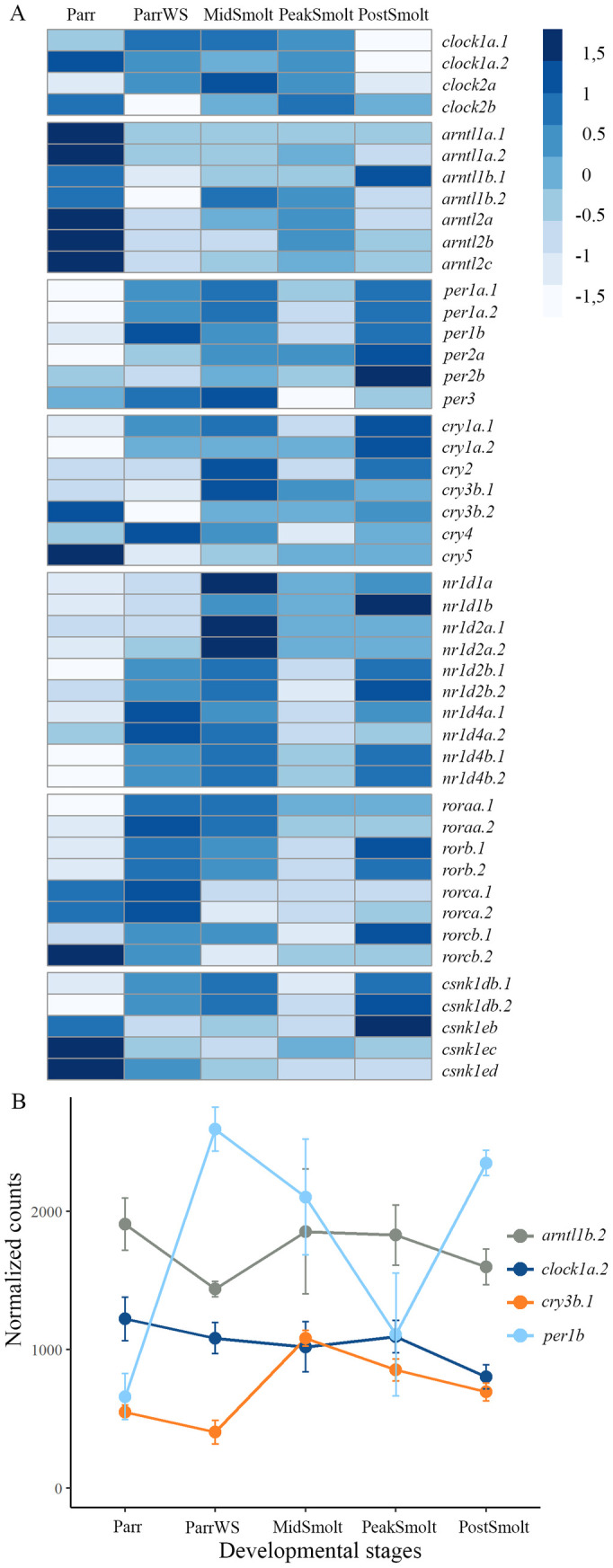
Expression profiles of the clock genes in the brain of Atlantic salmon. **A)** The heatmap of normalized counts during smoltification is scaled by row and shows a dynamic expression of the clock genes. **B)** Normalized counts with standard deviation are plotted for selected differentially expressed genes within each class of the core clock genes, *clock*, *arntl*, *cry* and *period*. The profiles show prominent differences between the genes within each class and developmental stages, e.g., *arntl1b* and *per1b* have opposite peaks and troughs at ParrWS, PeakSmolt and PostSmolt.

A total of 35 clock genes were among the DEGs detected in the brain sampled at the same time of the day during smoltification (see S5 Table for details on significant changes between timepoints). The heatmap of normalized counts of all clock genes shown in Fig 6A, reveal that several of the genes changed expression levels in the brain from Parr to ParrWS, when there was a change in the photoperiod from LD20:4 to LD12:12. Also, changes in expression levels of the clock genes were detected in ParrWS compared to MidSmolt, with the photoperiodic shift from LD 12:12 to LD24:0. Interestingly, the developmental stages MidSmolt, PeakSmolt and PostSmolt, all exposed to LD24:0, had differential expression of clock genes and analysis revealed nine DEGs comparing PostSmolt to PeakSmolt, with seven of the genes being upregulated in PostSmolt (S5 Table). Further, Fig 6B demonstrates detailed expression profiles of differentially expressed core clock genes, one member from each class, with expression profiles of all DEGs in each class in S6 Fig. Among the *clock* members, *clock1a.2* was the only DEG (S5 Table), with a slight decrease in expression towards the seawater phase (Fig 6B and S6A Fig). All members of the *arntl* class were differentially expressed during smoltification and in general the members had the highest expression level in Parr and were downregulated in ParrWS (Fig 6A, S6A Fig and S5 Table). Illustrated by *arntl1b.2* that had a decreased by approximately 0,5-fold between Parr and ParrWS, before a slight increase in expression level towards PeakSmolt, followed by a decrease in PostSmolt (Fig 6B). All *period* members, except *per2a* were among the DEGs and showed a similar trend in expression (S6B Fig and S5 Table). Highlighted by the *per1b* with the most prominent change, having an almost 2-fold increase between Parr and ParrWS, before a decrease of more than 1-fold between ParrWS and PeakSmolt and a subsequent 1-fold increase in expression level between PeakSmolt and PostSmolt (Fig 6B). Among members of the *cryptochrome* class, all genes except *cry2* were differentially expressed and *cry3b.1* had the greatest change in expression level, with an almost 1,5-fold increase between ParrWS and MidSmolt, followed by a decrease towards the seawater phase (Fig 6B, S6C Fig and S5 Table). In the *nr1d* class, all genes except *nr1d4a.*2 were differentially expressed (S5 Table). Notable, *nr1d1a*, *nr1d2a.2*, *nr1d4b.1* and *nr1d4b.2* had dynamic expression profiles that peaked at MidSmolt, decreased in PeakSmolt before increasing again in PostSmolt. For example, *nr1d4b.1* had an almost 1,5-fold increase in expression level between Parr and MidSmolt, before a 0,5-fold decrease between MidSmolt and PeakSmolt and a 0,5-fold increase between PeakSmolt and PostSmolt (S6D Fig). In the *ror* class, *roraa.1* and *roraa.2* had the highest expression level with a significant increase from Parr to ParrWS, that counted for an over 0,5-fold change between the two stages, and the two DEGs in the *cnsk* class had an increase in expression from the Parr to PostSmolt (S6 Fig and S5 Table). In addition, the distribution of normalized counts for clock genes was visualized by percentage to reveal the differential expression profile between the eye and brain during smoltification (S7 Fig). In difference from the nonvisual opsin genes, the expression levels of clock genes were more evenly distributed. The genes with highest percentage in both tissues were *per1b* (around 8% in the eye and 5% in the brain) and *rorcb.1* (6,5% in the eye and 6% in the brain) (S7 Fig).

**Fig 7:**
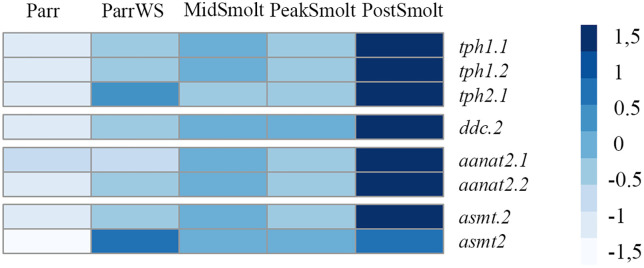
Expression levels of important genes in the melatonin synthesis in the brain of Atlantic salmon. The heatmap, scaled by row, shows the differentially expressed genes (DEGs) with a count more than 100 in the melatonin synthesis from tryptophan to melatonin, revealing in increased expression towards the seawater phase.

Further, melatonin is an important output of the circadian clock mechanism and genes coding for enzymes that dictate melatonin synthesis were analyzed. Most genes involved in the enzymatic steps were differentially expressed in the brain and had an upregulation from Parr to PostSmolts (Fig 7 and S5 Table). Interestingly, the two paralogues of the melatonin synthesizing enzyme arylalkylamine N-acetylserotonin 2 (*aanat2.1* and *aanat2.2*) were significantly upregulated by approximately 6,5-fold and 4-fold, respectively, from Parr to PostSmolt, where about 2-fold and 1,5-fold of the increase took place in the transition from PeakSmolt to PostSmolt (S5 Table). Also, two paralogues of the last enzyme in the synthesis of melatonin N-acetylserotonin O-methyltransferase (*asmt.2* and *asmt2*) had an approximately 4,5-fold and 1-fold, respectively, significant increase from Parr to PostSmolt (Fig 7 and S5 Table). The distribution of normalized counts for all important enzymes of the melatonin synthesis was visualized by percentage to reveal the differential expression profile between the eye and brain during smoltification (S8 Fig). Of notice, the expression levels of *aanat1* and *aanat2* were as previously reviewed [49,50] mainly distributed between the eyes and brain, respectively, with the three *aanat1* genes counting for over 38% of the important melatonin synthesizing enzymes in the eye, while *aanat2* counted for approximately 22% in the brain (S8 Fig).

### Dynamics of genes in the mammalian model for photoperiodism in the brain

**Fig 8:**
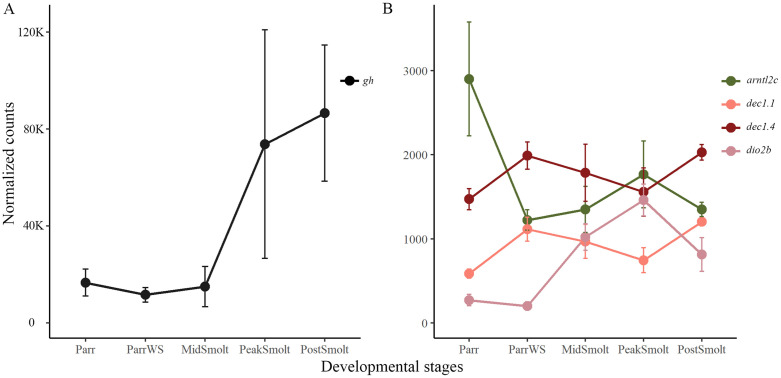
Growth hormone and selected genes in the model of photoperiodism. The graphs show normalized counts in the brain with standard deviation, illustrating the dynamics through smoltification. **A)** The growth hormone (*gh*) increased towards the seawater phase, with a great increase from MidSmolt to PeakSmolt. **B)** The *dec1* genes are represented by the expression patterns of *dec1.1* and *dec1.4*, showing the similar expression pattern between paralogues, while the *arntl2* genes are represented by *arntl2c*, showing opposing expression peaks at the different sampling points through smoltification. The expression level of *dio2b* was high at PeakSmolt.

The expression profiles of the pituitary specific growth hormone (*gh*) and important elements of the mammalian model for photoperiodism were analyzed in the period of parr-smolt transformation (Fig 8 and S5 Table showing differentially expressed genes). A large increase in the expression level of *gh* was shown between MidSmolt and PostSmolt, with an over 2-fold increase between MidSmolt and PeakSmolt, indicating of a proper parr-smolt transformation (Fig 8A). The genes in the photoperiodic switch, *arntl2* and *dec1*, together with markers for summer (*eye absent*, *eya3*) and winter (*chromogranin A*, *chga*) physiology were studied [36]. In addition, the expression patterns of the *deiodinases*, the gatekeepers for controlling the availability of active or inactive thyroid hormone, that are implied to be involved in developmental and seasonal physiology of salmonids [51,52], were studied. The expression levels of all genes were plotted in S9 Fig and selected DEGs are shown in Fig 8B. Among the *dec1* genes, three genes (*dec1.1*, *dec1.3* and *dec1.4*) were significantly upregulated in ParrWS compared to Parr, while the three *arntl2* genes were downregulated in the same comparison (S5 Table). The photoperiodic switch is illustrated by plotting *arntl2c* and two *dec1* genes (*dec1.1* and *dec1.4*, illustrating the similar expression pattern between paralogues) (Fig 8B). Comparing Parr and ParrWS *dec1.1* and *dec1.4* had an approximately 1-fold and 0,5-fold upregulation, respectively, while *arntl2c* had a 1-fold decrease. Further, *dec1.1* was significantly downregulated between ParrrWS and PeakSmolt, while the *arntl2* genes were upregulated comparing PeakSmolt and ParrWS (S5 Table and S9 Fig). The marker for winter physiology, *chga*, was upregulated in ParrWS compared to Parr and downregulated in PeakSmolt compared to ParrWS, while the two paralogues of *eya3*, the marker for summer physiology, were not differentially expressed (S5 Table and S9 Fig). Note that the level of thyroid-stimulating hormone (*tsh*β*b*) expressed in the pituitary had a low expression level throughout smoltification, counting less than 10 normalized counts in all samples. Among the *deiodinases*, *dio2b* was differentially expressed through smoltification (S5 Table), with high expression levels at PeakSmolt after an approximately 2,5-fold increase in expression from Parr to PeakSmolt, while both *dio2a* and *dio3a2* were lowly expression in the brain (S9 Fig).

## Discussion

The photoreceptive capacity of an organism resides within the array of opsins in the genome, spectral properties of the photopigments and the dynamic expression of opsins at different life stages and light environments [4]. This study investigates the expression profiles of opsin genes in the eye and brain of Atlantic salmon during smoltification to reveal shifts in expression through this developmental transition with a change from freshwater to seawater, marking a change in light environment under natural conditions. In addition, the circadian clock system that is entrained by light inputs has been studied, including important elements of the melatonin synthesis, a major output of the clock mechanism. The results provide a comprehensive understanding of the dynamics of the photoreceptive system and clock mechanism when the habitat is changed from river to ocean where the blue part of the spectrum penetrates deepest in the water column. However, it is important to recognize that the change in transcript level does not necessarily translate into functional changes at the protein or physiological level. The transcriptomic profile of the brain during smoltification coincides with expression patterns in the mammalian model of photoperiodism driving summer and winter physiology, supporting a common photoperiodic regulation of seasonality in vertebrates.

### The tandem duplicated visual opsin genes have shifts in expression during smoltification

The transcriptomic profile of visual opsin genes in Atlantic salmon larvae and alevins confirmed previous findings, showing that the visual opsins are expressed before first feeding, with a duplex retina dominated by rhodopsin expression from the start [11,53]. The sampling points before (379 dd) and after hatching (555 dd), showed that the opsins were expressed after hatching with an increased level towards first feeding. All opsin classes were present before first feeding, including the four members of the *rh2* class and two of the members in the *lws* class, providing the alevin with tetrachromatic vision. In accordance with previous studies, there was a decline in expression of *sws1−1* in the parr-smolt transformation, causing the vision to become trichromatic towards the seawater phase [26]. Interestingly, the tandem duplicated medium-wavelength-sensitive and long-wavelength-sensitive opsin genes showed a dynamic expression during smoltification, not previously described. In freshwater, *rh2−3* and *rh2−4* dominated the expression among the green opsins, but at PeakSmolt there was a shift in expression to *rh2−1*, while the *rh2−2* had low expression through smoltification. The change to *rh2−1* counted for almost 40 nm shifts towards shorter wavelengths based on the predicted absorbance maxima (i.e., λ_max_ values), coinciding well with the change in light environment from river and ocean. Further, the tandem duplicated *lws1* and *lws2* counted for most of the expression in the *lws* class, with the *lws1* appearing towards the seawater phase. Note that the changes in green and red opsins occurred under equal spectrum and intensities of light in this experiment and further studies are needed to verify additional changes in the visual system related to direct adaptation to the natural light in the marine environment. This will help to fully understand the photoreceptive adaptation and tuning under this life history transition. Thus, it is difficult to distinguish if the change in opsin gene expression is due to shifts in photic environment or an effect of the developmental transition. Differential expression pattern of the green and red opsin genes is well described in fish and has been shown both in time and space [20,27,54], although not described in a developmental transition such as smoltification. For example, during zebrafish development *rh2−1* and *rh2−2* were expressed earlier than *rh2−3* and *rh2−4* and in adult zebrafish *rh2−1* and *rh2−2* were located dorsally, while *rh2−3* was expressed in a restricted area ventrally, next to the ventral-most *rh2−4* expression. In addition, *lws1* was found in the ventral region and *lws2* in the dorsal region, and for both classes the paralogues with the highest absorbance maximum were expressed in the ventral region [20,28]. Zebrafish have been used as models for understanding the differential regulation of tandemly arrayed genes, and the varying spatial distribution of green and red opsins in the zebrafish eye has been shown to be determined by the gene order, proximal regulator regions and a common locus control region [28,55]. Further, the thyroid hormone was shown to be involved in regulation of green and red opsins in zebrafish, as abundance of TH shifts the expression to ventrally expressed opsins with sensitivities in the longer wavelengths (*rh2−3*, *rh2−4* and *lws1*) [56]. Similarly, it has been shown that retinoic acid switches the expression from *lws2* to *lws1* and that the RA signaling domain coincides with a ventral zone of *lws1* expression in zebrafish [57]. Analysis of the TH metabolism in the salmon eye during smoltification revealed that *dio2b* converting the inactive T4 to the active T3 [58] was upregulated at PeakSmolt. While the *dio3a2* involved in the inactivation of TH by converting T4 to T3 and degradation of T3 to T2 [58] had a peak at MidSmolt. The upregulation of *dio2b* at PeakSmolt, promoting active TH, coincided with the upregulation of *lws1* having the highest absorbance maximum. However, among the green opsins *rh2−3* and rh*2−4* decreased and *rh2−1* increased at PeakSmolt, supporting a shift to shorter wavelengths, and expression of a paralogue known to be dorsally expressed in zebrafish. Evaluating the expression of important enzymes in the conversion of retinol to retinoic acid [59] showed an upregulation towards the seawater phase, with the retinaldehyde dehydrogenase (*aldh1a1* and *aldh1a3.2*) peaking at PeakSmolt coinciding with the *lws1* expression. However, a member of the cryptochrome P450 class involved in the degradation of RA [59] was peaking at PostSmolt, suggesting that a ventralizing signal of RA in PostSmolt was suppressed. Taken together, the conserved synteny of the tandem duplicated green and red opsins [60] and differential expression of the opsins suggest a similar regulation mechanism in Atlantic salmon as in zebrafish. Thus, elucidating the special distribution of opsins in the retina during smoltification would give an understanding of the dorsal-ventral patterning of opsins in salmon.

### Dynamic expression of a *rgr* class member and the pineal specific exorhodopsin

Only a few nonvisual opsins showed a substantial shift in expression in the eye and brain during smoltification. In the eye, *rgrb2* was highly expressed during smoltification but with a decrease in expression in PeakSmolt, while *exorh* showed an increased expression from the parr to the seawater phase in the brain. Comparing the expression of the nonvisual opsins in the eye and brain showed that members of the *rgr* class dominated in both tissues and the differential expression between *rgrb1* and *rgrb2* in the brain and eye, might account for a subfunctionalization of these salmonid specific paralogues. Members of the *rgr* class are suggested to be both opsins and photoisomerases and have been shown to be highly expressed in retinal pigmented epithelium of the eye, preferably binding to all-*trans* retinal [16]. A dynamic expression of *rgrb2* in the eye during smoltification might be associated with shifts in expression of visual opsin genes probably with a function as a photoisomerase, photo-converting the chromophore from all-*trans* to 11-*cis* retinal [61]. The pineal-specific opsin *exorh*, expressed with a daily rhythmicity in zebrafish is suggested be involved in the melatonin synthesis as knockdown morpholino studies with *exorh* decreased the melatonin synthesizing enzyme (*aanat2*) [62]. Also, single cell RNA sequencing of the pineal organ of zebrafish has shown that the pineal photoreceptor cells express both *aanat2* and the last enzyme in the synthesis of melatonin the N-acetylserotonin O-methyltransferase (*asmt*) [63]. Interestingly, the great upregulation of *exorh* during smoltification corresponded with an upregulation of genes in the synthesis of melatonin and might be related to growth of the pineal organ to provide a greater light sensitivity in an oceanic environment.

### Clock genes showed dynamic expressions in the parr-smolt transformation

A total of 35 clock genes were differentially expressed in the brain with a dynamic expression during smoltification, including members of the core clock and the stabilizing loop. Similarly, many clock genes were shown to be dynamically expressed in the gills during smoltification, with a subset of clock genes suggested to be associated with the physiological preparation of seawater migration, independent of circadian regulation [38]. Further, earlier studies in the parr brain have shown daylength dependent expressions, by differences in expression between fish exposed to long or short photoperiods, with a 4 hour shift in acrophase for *cry2*, while other components of the core clock became arrhythmic under long photoperiods [37]. In the current study, analysis indicates that the differential expression was related both to daylength and the developmental transition from freshwater to seawater, as dynamic expression was detected between stages with different photoperiods, but also between stages in constant light. However, brains were sampled at the same time point at daytime at each stage and the change in expression between stages exposed to different photoperiods might be a result of shifted acrophase and should be interpreted with caution. For example, parr under LD12:12 with lights on at 08:00, had an acrophase at 08:30 for *per1b* [64] and the great shift in expression of *per1b* between Parr (LD 20:4 lights on at 04:00, ZT8) and ParrWS (LD12:12 lights on 08:00, ZT4) in the current study might be a result of a shift in acrophase due to changed light period. However, in MidSmolt, PeakSmolt and PostSmolt the fish were all held under continuous light, but three clock genes were significantly downregulated from MidSmolt to PeakSmolt and as illustrated by the *per1b* expression, a total of nine clock genes were either up- or downregulated between PeakSmolt and PostSmolt. The change in expression of clock genes under constant light conditions, might account for a shift related to the developmental transition from freshwater to seawater.

### Photoperiodic regulation of smoltification

The level of plasma growth hormone is known to increase in Atlantic salmon as a response to a longer photoperiod in the spring [35,65,66]. The increase has been shown to be significant 20 days after the switch to longer days, consistent with a prior elevation of the *tsh*β*b*, followed by an increase in *dio2b* in the brain [35]. However, the expression level of *gh* in the pituitary was not significantly increased after 20 days and has been suggested to be elevated later to compensate for a drop in the pituitary *gh* content [35,67]. The present results are consistent with these suggestions as the mRNA level of *gh* first increased at PeakSmolt, approx. 44 days after the switch to longer days. The transcriptome was also analyzed for elevation of *tsh*β*b* and *dio2b* during smoltification and while *dio2b* increased in the brain at PeakSmolt as reported previously [35,52], the expected elevated level of *tsh*β*b* at MidSmolt was not found. Though, the expression of *tsh*β*b* in the few cells of the dorsal pituitary, near the pituitary stalk [68], is likely to have be masked by sequencing of the whole brain, and future studies should analyze the brain and pituitary separately. Factors in the mammalian model of photoperiodism were also analyzed [36] and *chga* the marker for winter physiology peaked in ParrWS while the two *eya3* paralogues, marking long days in mammals, were not differentially expressed in the brain during smoltification. Interestingly, genes involved in the circadian clock mechanism suggested to function as a photoperiodic switch between the winter and summer physiology in mammals, showed differential expression during smoltification. The *dec1* paralogues promoting winter physiology were significantly upregulated in ParrWS, coinciding with downregulation of *arntl2* paralogues, followed by upregulation of *arntl2* paralogues at PeakSmolt. Taken together, these results support a common photoperiodic regulation of seasonality in vertebrates and highlight a need for an in dept study of the pituitary transcriptome during smoltification.

## Supporting information

S1 Table Normalized counts of the eyes through the parr-smolt transformation. (XLSX)

S2 Table Normalized counts of the brains through the parr-smolt transformation. (XLSX)

S3 Table Annotated Atlantic salmon genes v3. Annotations of nonvisual opsins, genes in the metabolism of thyroid hormone and retinoic acid, and genes in the mammalian model of photoperiodism.
(XLSX)

S4 Table Differentially expressed visual opsins, nonvisual opsins and factors related to thyroid hormone (TH) and retinoic acid (RA) in the eye through smoltification, with an adjusted with an adjusted p-value set to < 0.05. All stages are compared to each other and the differentially expressed genes for each comparison are listed. In the comparisons the stage put lastly is the control, and the up- or downregulation notes are reflecting the expression of the first mentioned stage compared to the control.
(XLSX)

S5 Table Differentially expressed nonvisual opsins, clock genes, genes in the melatonin synthesis and in the model of photoperiodism including growth hormone (*gh*) in the brain through smoltification, with an adjusted with an adjusted p-value set to < 0.05. All stages are compared to each other and the differentially expressed genes for each comparison are listed. In the comparisons the stage put lastly is the control, and the up- or downregulation notes are reflecting the expression of the first mentioned stage compared to the control.
(XLSX)

S1 Fig Experimental design and sampling. Atlantic salmon were reared from fertilization to one month in seawater (557 days in total). The embryonic stages and alevins were sampled in [17] but illustrated here. At first feeding, the fish were transferred to feeding tanks and the feeding fry were reared to the parr stage under LD20:4. Parr were sampled approx. nine weeks before turning the light period to winter signal (WS) LD12:12 for six weeks. ParrWS were sampled the last day of the winter signal and the light was turned to LD24:0 the next day. After turning to continuous light, MidSmolt were sampled 22 days and PeakSmolt 51 days later, respectively. The fish were transferred to seawater 16 days after sampling of PeakSmolt and reared for a month in seawater before the last sampling at PostSmolt.
(TIF)

S2 Fig Weight, length and condition factor through smoltification. Each parameter is plotted with standard deviation as follows A) weight B) length C) condition factor (K-factor).
(TIF)

S3 Fig Principal Component Analysis (PCA) plots. A) The variability of eye samples by stage shows that the stages were quite distinct, with ParrWS being the most separated. B) The variability of brain samples by stage shows that the stages, other than Parr, were quite intermingled.
(TIF)

S4 Fig Genes involved in the metabolism of thyroid hormone (TH) and retinoic acid (RA). All normalized counts in the eye are plotted with standard deviation. A) The expression of two of the deiodinase members increased, specifically, *dio2b* increased towards PeakSmolt while *dio3a2* peaked at MidSmolt, while *dio2a* showed a very low expression. B) All three retinol dehydrogenase (*rdh*) genes were expressed in the eye, with *rdh10a2* being the predominant member with increased expression levels towards the seawater phase. C) Retinaldehyde dehydrogenases expression levels were generally stable throughout smoltification, with dynamic expression patterns observed for both *aldh1a* and *aldh1a3.2*, with highest expression evident at PeakSmolt. D) A member of the cryptochrome P450 class, specifically *cyp26b1.1*, peaked in expression at PostSmolt, while all other *cyp26* members analyzed were expressed lowly throughout all four developmental stages.
(TIF)

S5 Fig Differentially expressed nonvisual opsin genes in the eye of Atlantic salmon. The graphs show, with standard deviation, normalized counts of differentially expressed nonvisual opsin genes during smoltification. A) Members of the retinal G protein-coupled receptor (*rgr*) opsin family, with great variation in *rgrb2* and with the lowest expression at PeakSmolt. B) The genes parapinopsinb (*ppb*) and vertebrate ancient opsin (*va1b*) were differentially expressed. C) Among the *opn3* and *tmtopsin* genes, only *tmtopsin1a1* was differentially expressed. D) The only differentially expressed gene in the *opn4* class was *opn4m1a1* with highest expression in PeakSmolt. E) Among the genes in the *opn5* to *opn9* classes, three genes were differentially expressed, *opn6a1* had the highest expression level, with a decreasing expression level towards the seawater phase.
(TIF)

S6 Fig Differentially expressed clock genes in the brain of Atlantic salmon. The graphs show normalized counts of differentially expressed clock genes with standard deviation at five sampling points during smoltification. A) The differentially expressed members of the *clock* and *arntl* classes. B) In the *period* class, *per1b* had a dramatic change in expression level, peaking at ParrWS and PostSmolt. C) Among the *cryptochromes*, *cry3b.1* had the lowest expression at ParrWS and peaked at MidSmolt. D) Many of the genes in the *nr1d* class were differentially expressed, several had a peak at MidSmolt followed by a trough in PeakSmolt. E) In the Ror class, *roraa.1* and *roraa.2* increased in expression levels from Parr to ParrWS. F) Two genes were differentially expressed in the *csnk* class, with an increase in expression from Parr to PostSmolt.
(TIF)

S7 Fig Distribution of clock genes in the eye and brain of Atlantic salmon. Bar chart of the clock genes plotted by percentage in the eye and brain. The total number of normalized counts at the four sampling points (ParrWS to PostSmolt) for the clock genes was set to 100 percent for each tissue and the distribution among the different genes was plotted back-to-back. The plot reveals that the distribution among the clock genes were quite even, genes in each class were expressed both in the brain and eye.
(TIF)

S8 Fig Distribution of important genes in the synthesis of melatonin in the eye and brain of Atlantic salmon. Bar chart of the important genes dictating the melatonin synthesis plotted by percentage in the eye and brain. The total number of normalized counts at the four sampling points (ParrWS to PostSmolt) for the genes was set to 100 percent for each tissue and the distribution among the different genes was plotted back-to-back. Members of *aanat1* class were expressed in the eye while the two *aanat2* paralogues were mainly expressed in the brain.
(TIF)

S9 Fig Expression patterns of selected genes in the mammalian model of photoperiodism. Normalized counts in the brain are plotted with standard deviation. A) The *dec1* genes had a peak in ParrWS and in PostSmolts. B) The *arntl2a* and *arntl2c* had a similar expression pattern, with a decrease in expression from Parr to ParrWS and a subsequent increase in PeakSmolt, while *arntl2b* was lowly expressed. C) The marker for winter physiology *chga* had a peak in ParrWS and in PostSmolt while the two *eya3* paralogues (summer physiology) were not differentially expressed. D) The *dio2b* had a great increase in expression towards PeakSmolt, while *dio2a* and *dio3b2* had low expression.
(TIF)
