# Supplementary figures and images for: Developmental transition of visual and nonvisual photoreception and circadian clock during smoltification in the eye and brain of Atlantic salmon
Source: PLoS One. 2026 May 21;21(5):e0349748. doi: 10.1371/journal.pone.0349748 (PMC13193417; doi:10.1371/journal.pone.0349748)

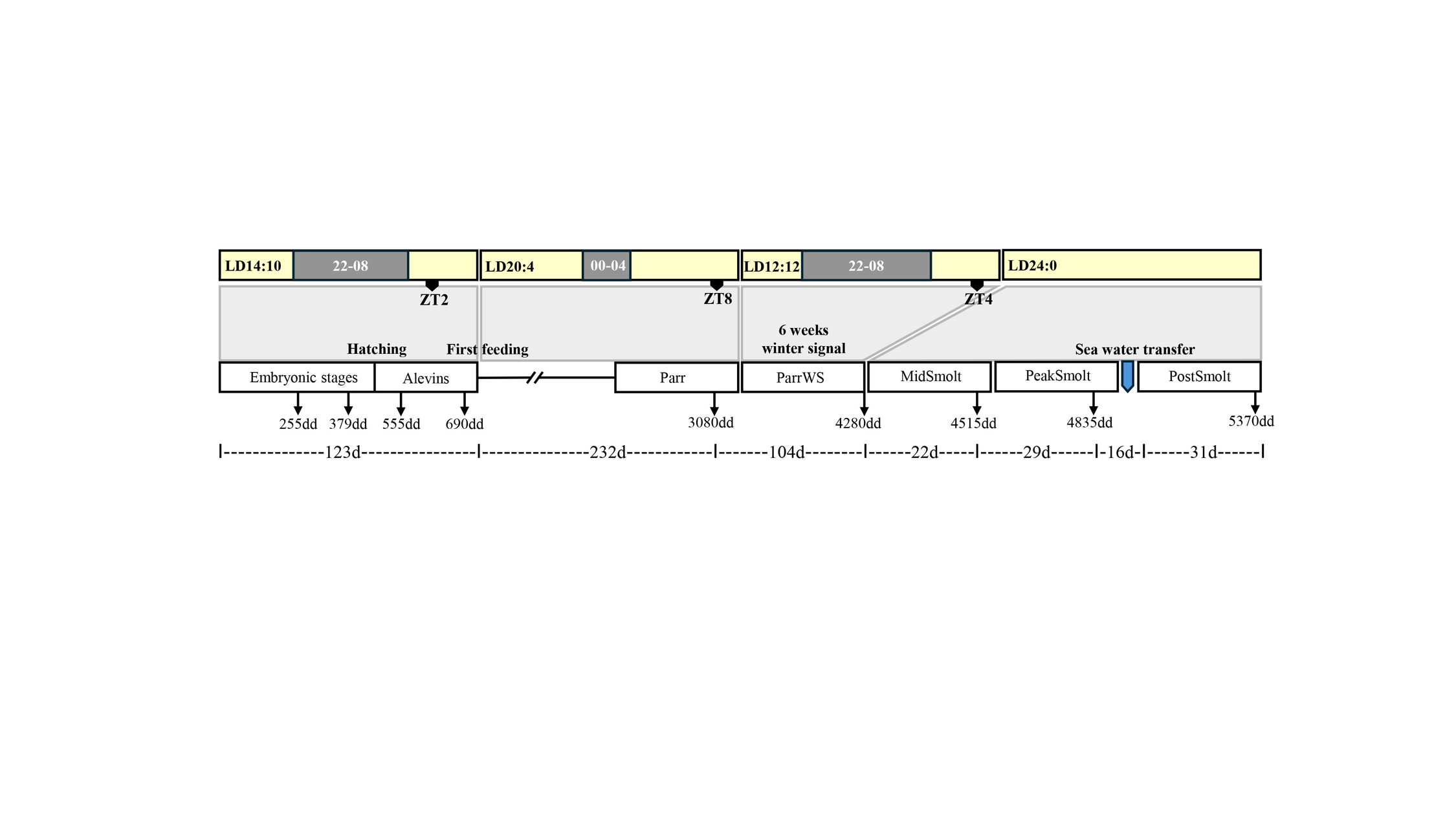

Supplement: S1 Fig — Atlantic salmon were reared from fertilization to one month in seawater (557 days in total). The embryonic stages and alevins were sampled in [17] but illustrated here. At first feeding, the fish were transferred to feeding tanks and the feeding fry were reared to the parr stage under LD20:4. Parr were sampled approx. nine weeks before turning the light period to winter signal (WS) LD12:12 for six weeks. ParrWS were sampled the last day of the winter signal and the light was turned to LD24:0 the next day. After turning to continuous light, MidSmolt were sampled 22 days and PeakSmolt 51 days later, respectively. The fish were transferred to seawater 16 days after sampling of PeakSmolt and reared for a month in seawater before the last sampling at PostSmolt. (TIF) [file pone.0349748.s006.tif]

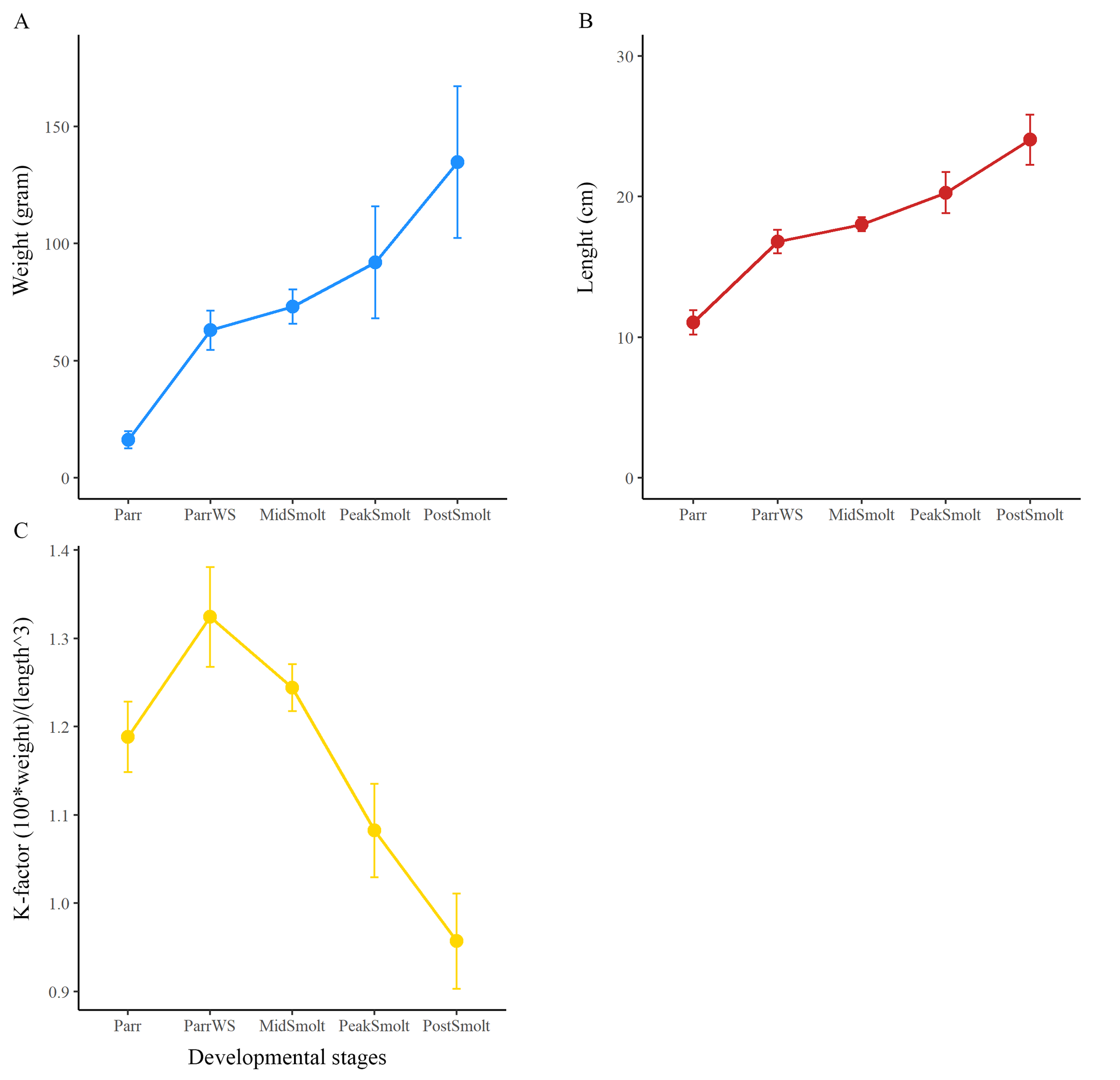

Supplement: S2 Fig — Each parameter is plotted with standard deviation as follows A) weight B) length C) condition factor (K-factor). (TIF) [file pone.0349748.s007.tif]

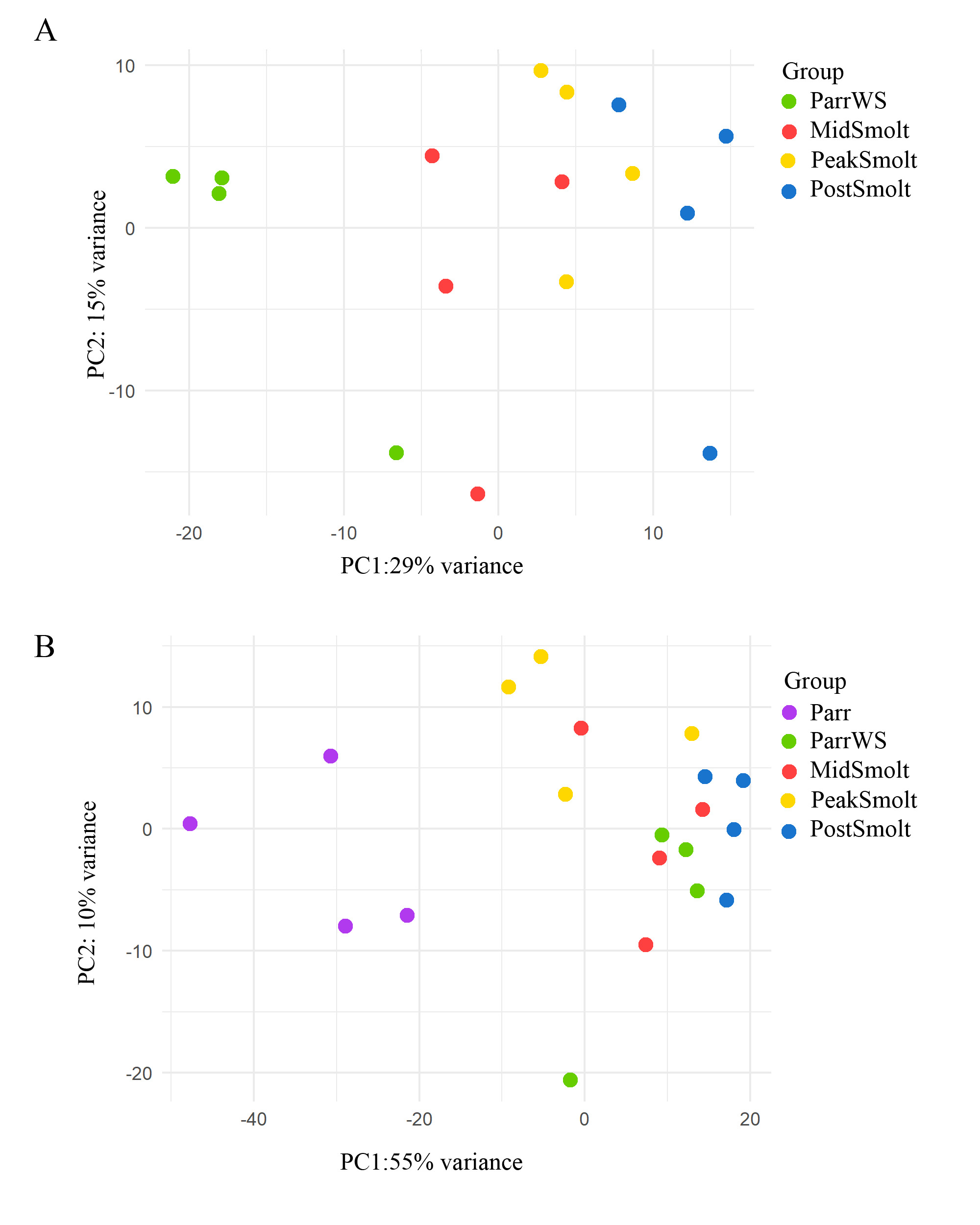

Supplement: S3 Fig — A) The variability of eye samples by stage shows that the stages were quite distinct, with ParrWS being the most separated. B) The variability of brain samples by stage shows that the stages, other than Parr, were quite intermingled. (TIF) [file pone.0349748.s008.tif]

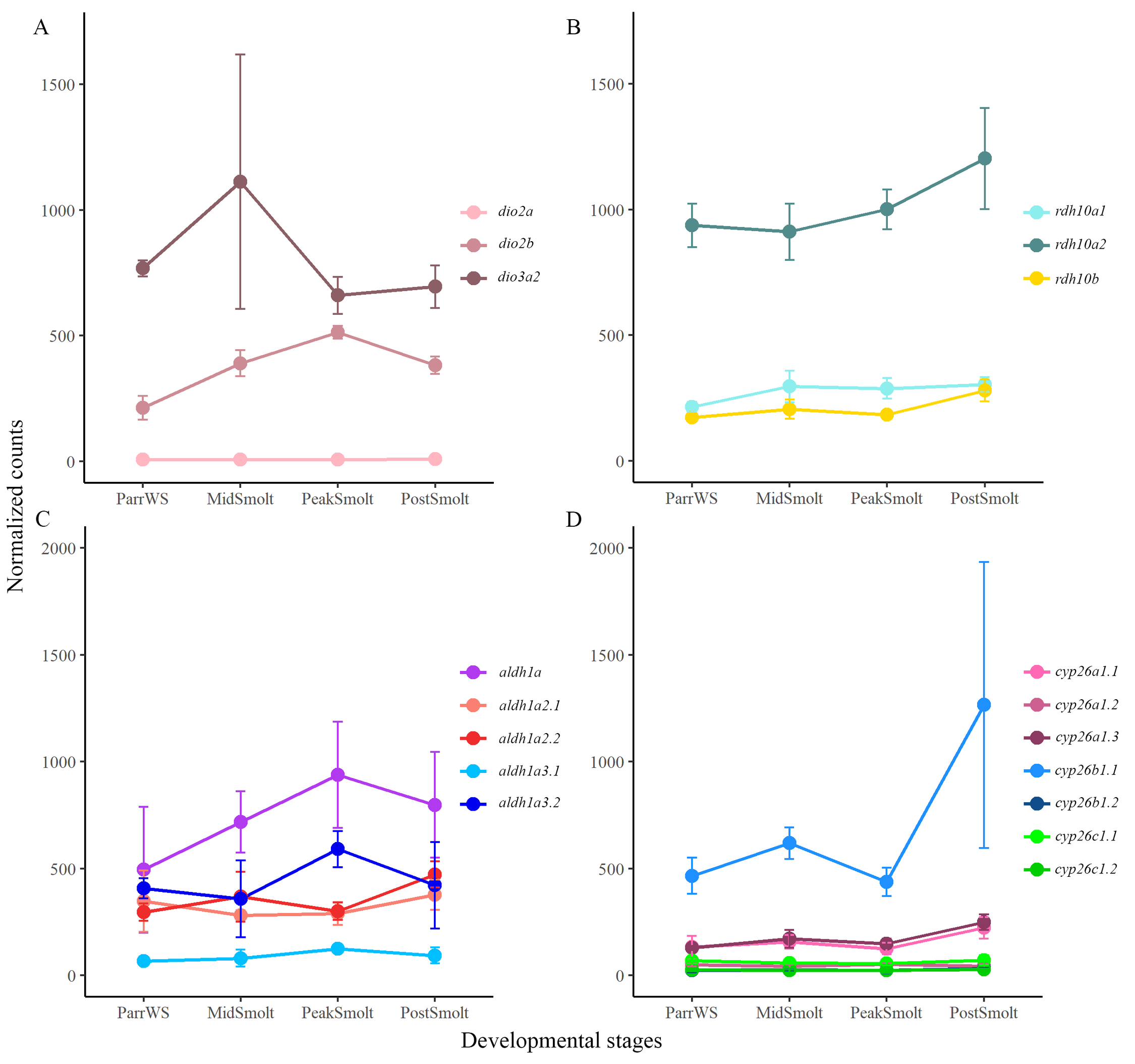

Supplement: S4 Fig — All normalized counts in the eye are plotted with standard deviation. A) The expression of two of the deiodinase members increased, specifically, dio2b increased towards PeakSmolt while dio3a2 peaked at MidSmolt, while dio2a showed a very low expression. B) All three retinol dehydrogenase (rdh) genes were expressed in the eye, with rdh10a2 being the predominant member with increased expression levels towards the seawater phase. C) Retinaldehyde dehydrogenases expression levels were generally stable throughout smoltification, with dynamic expression patterns observed for both aldh1a and aldh1a3.2, with highest expression evident at PeakSmolt. D) A member of the cryptochrome P450 class, specifically cyp26b1.1, peaked in expression at PostSmolt, while all other cyp26 members analyzed were expressed lowly throughout all four developmental stages. (TIF) [file pone.0349748.s009.tif]

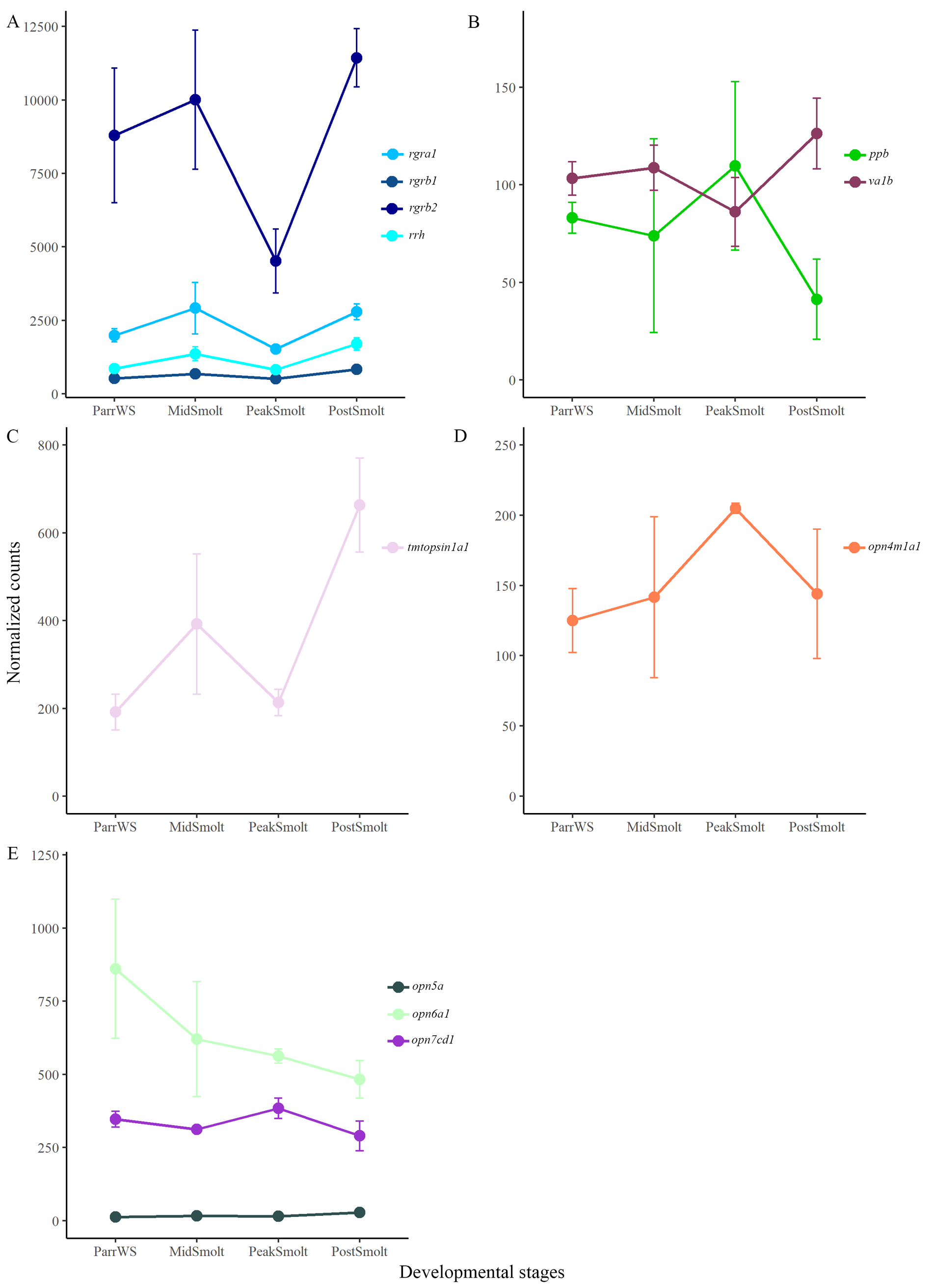

Supplement: S5 Fig — The graphs show, with standard deviation, normalized counts of differentially expressed nonvisual opsin genes during smoltification. A) Members of the retinal G protein-coupled receptor (rgr) opsin family, with great variation in rgrb2 and with the lowest expression at PeakSmolt. B) The genes parapinopsinb (ppb) and vertebrate ancient opsin (va1b) were differentially expressed. C) Among the opn3 and tmtopsin genes, only tmtopsin1a1 was differentially expressed. D) The only differentially expressed gene in the opn4 class was opn4m1a1 with highest expression in PeakSmolt. E) Among the genes in the opn5 to opn9 classes, three genes were differentially expressed, opn6a1 had the highest expression level, with a decreasing expression level towards the seawater phase. (TIF) [file pone.0349748.s010.tif]

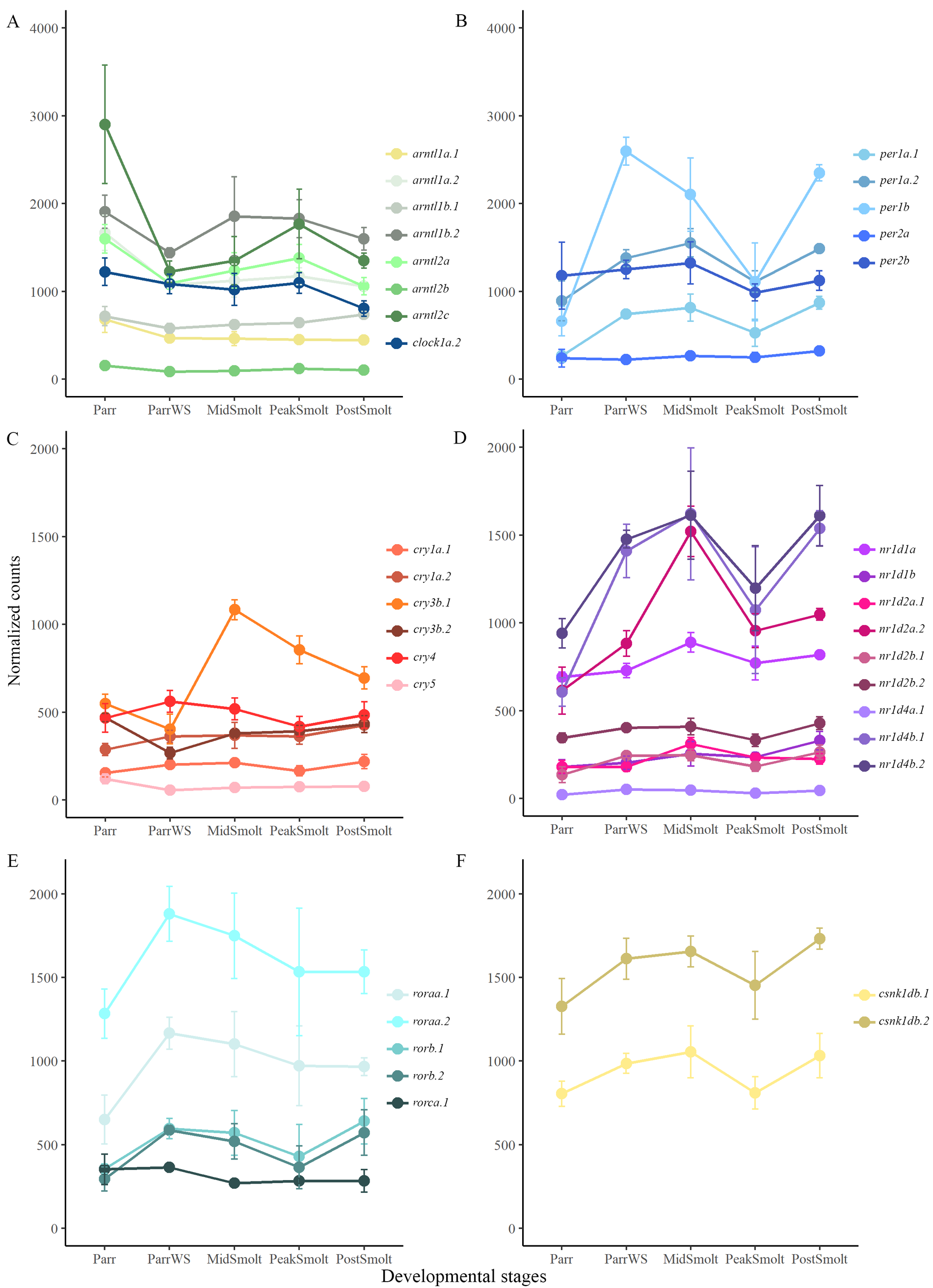

Supplement: S6 Fig — The graphs show normalized counts of differentially expressed clock genes with standard deviation at five sampling points during smoltification. A) The differentially expressed members of the clock and arntl classes. B) In the period class, per1b had a dramatic change in expression level, peaking at ParrWS and PostSmolt. C) Among the cryptochromes, cry3b.1 had the lowest expression at ParrWS and peaked at MidSmolt. D) Many of the genes in the nr1d class were differentially expressed, several had a peak at MidSmolt followed by a trough in PeakSmolt. E) In the Ror class, roraa.1 and roraa.2 increased in expression levels from Parr to ParrWS. F) Two genes were differentially expressed in the csnk class, with an increase in expression from Parr to PostSmolt. (TIF) [file pone.0349748.s011.tif]

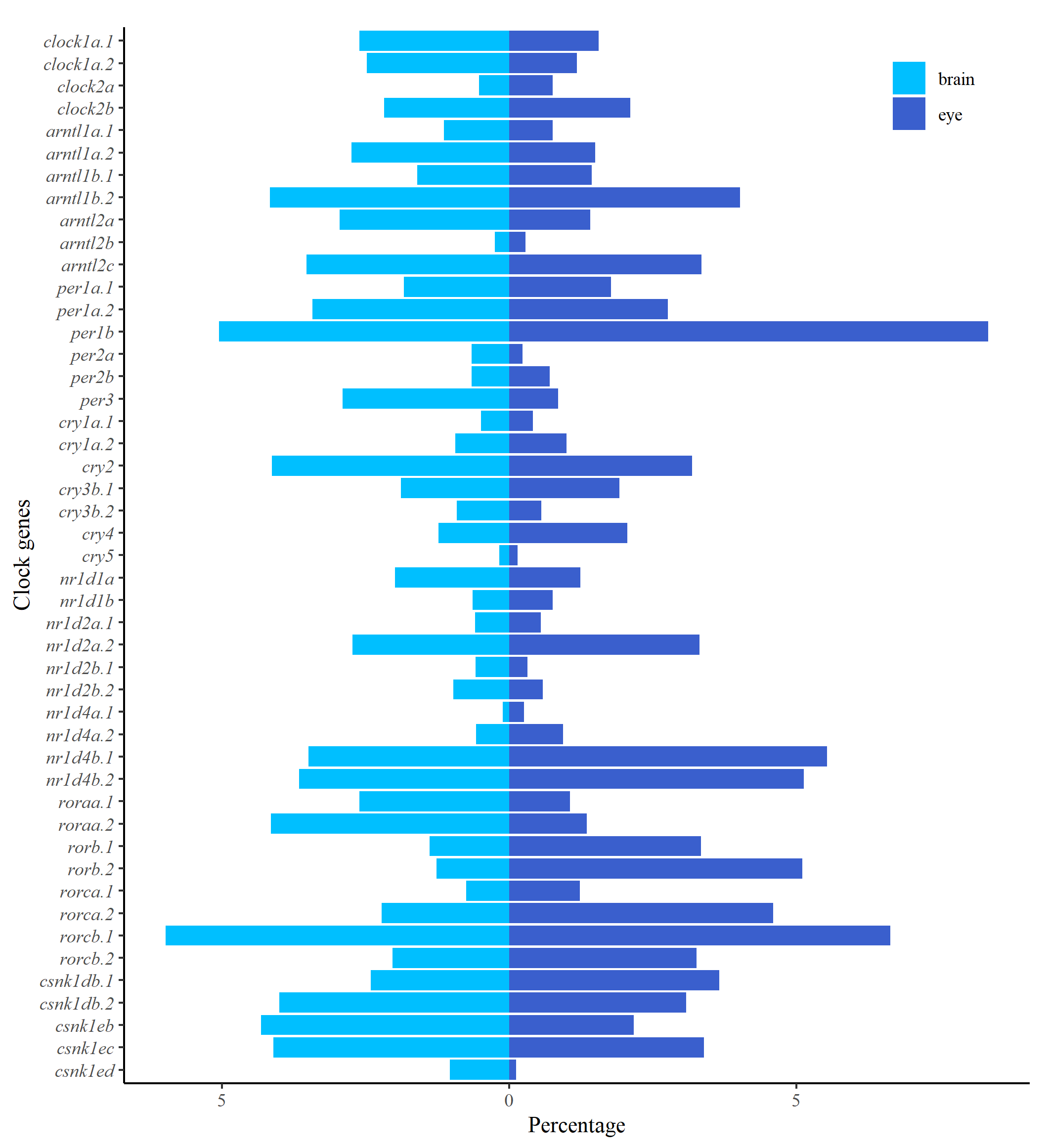

Supplement: S7 Fig — Bar chart of the clock genes plotted by percentage in the eye and brain. The total number of normalized counts at the four sampling points (ParrWS to PostSmolt) for the clock genes was set to 100 percent for each tissue and the distribution among the different genes was plotted back-to-back. The plot reveals that the distribution among the clock genes were quite even, genes in each class were expressed both in the brain and eye. (TIF) [file pone.0349748.s012.tif]

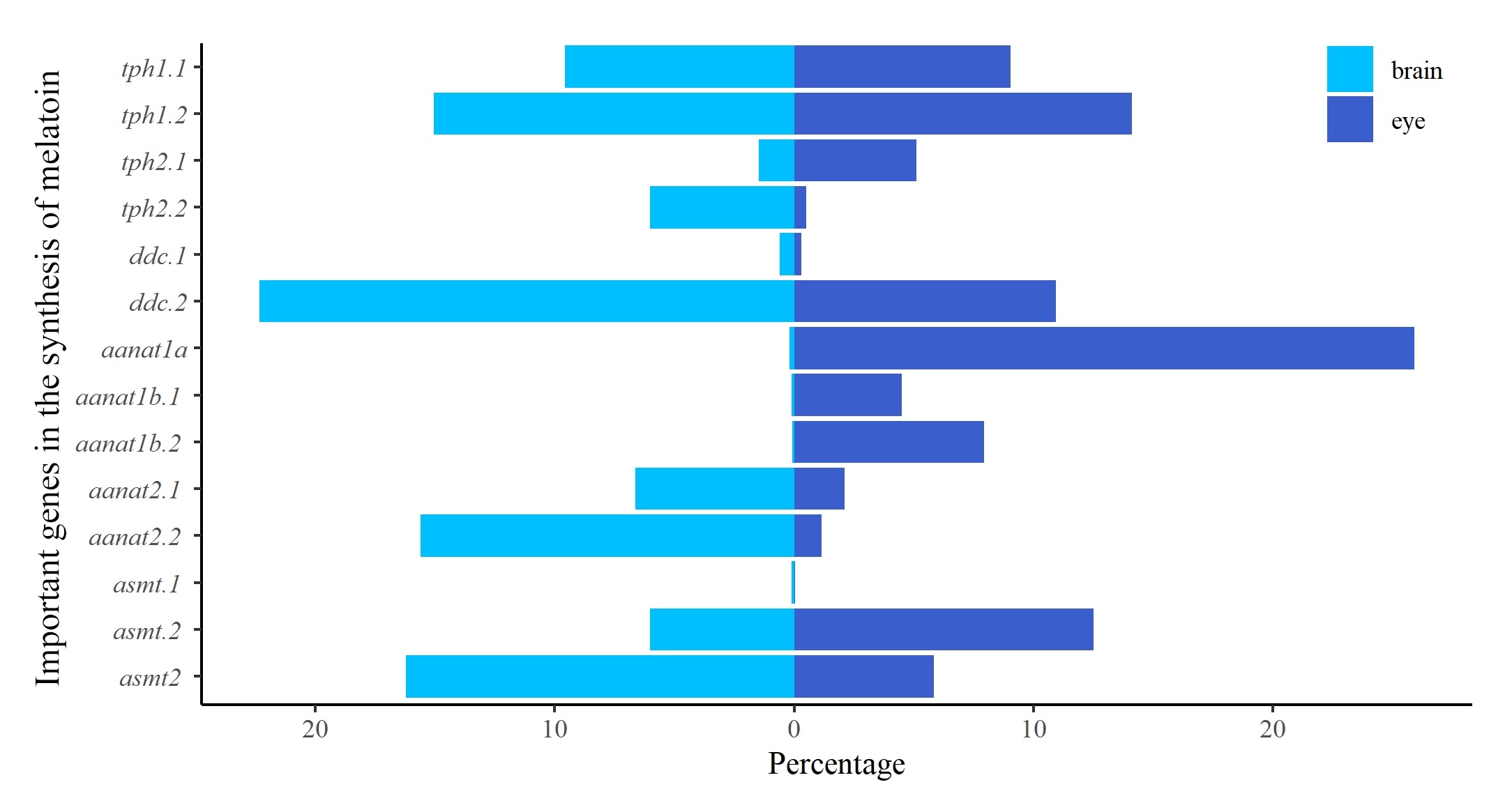

Supplement: S8 Fig — Bar chart of the important genes dictating the melatonin synthesis plotted by percentage in the eye and brain. The total number of normalized counts at the four sampling points (ParrWS to PostSmolt) for the genes was set to 100 percent for each tissue and the distribution among the different genes was plotted back-to-back. Members of aanat1 class were expressed in the eye while the two aanat2 paralogues were mainly expressed in the brain. (TIF) [file pone.0349748.s013.tif]

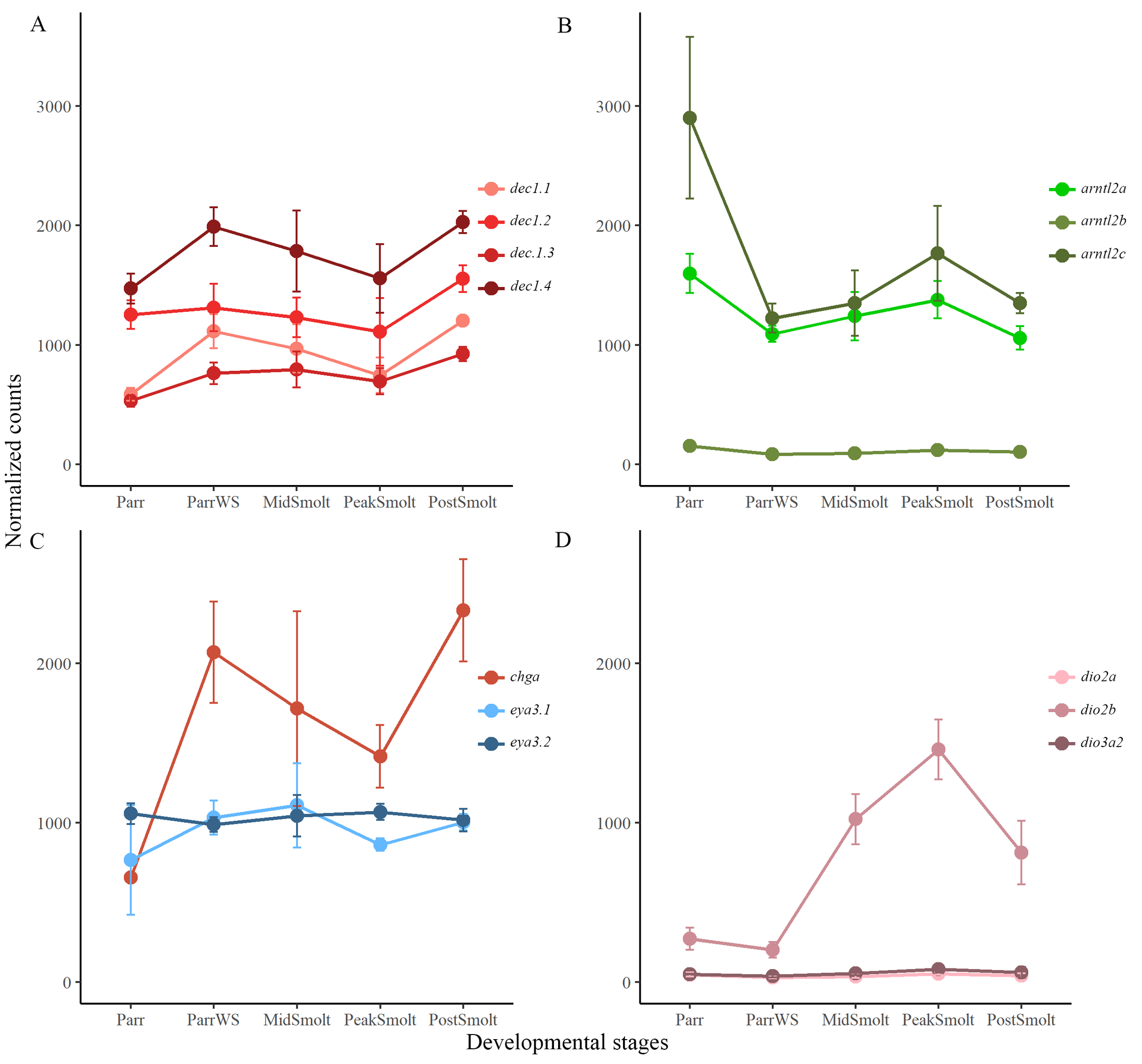

Supplement: S9 Fig — Normalized counts in the brain are plotted with standard deviation. A) The dec1 genes had a peak in ParrWS and in PostSmolts. B) The arntl2a and arntl2c had a similar expression pattern, with a decrease in expression from Parr to ParrWS and a subsequent increase in PeakSmolt, while arntl2b was lowly expressed. C) The marker for winter physiology chga had a peak in ParrWS and in PostSmolt while the two eya3 paralogues (summer physiology) were not differentially expressed. D) The dio2b had a great increase in expression towards PeakSmolt, while dio2a and dio3b2 had low expression. (TIF) [file pone.0349748.s014.tif]
